# Loss of phosphatase CTDNEP1 potentiates aggressive medulloblastoma by triggering MYC amplification and genomic instability

**DOI:** 10.1038/s41467-023-36400-8

**Published:** 2023-02-10

**Authors:** Zaili Luo, Dazhuan Xin, Yunfei Liao, Kalen Berry, Sean Ogurek, Feng Zhang, Liguo Zhang, Chuntao Zhao, Rohit Rao, Xinran Dong, Hao Li, Jianzhong Yu, Yifeng Lin, Guoying Huang, Lingli Xu, Mei Xin, Ryuichi Nishinakamura, Jiyang Yu, Marcel Kool, Stefan M. Pfister, Martine F. Roussel, Wenhao Zhou, William A. Weiss, Paul Andreassen, Q. Richard Lu

**Affiliations:** 1 Brain Tumor Center, Division of Experimental Hematology and Cancer Biology, Cincinnati Children’s Hospital Medical Center, Cincinnati, OH 45229 USA; 2 Key Laboratory of Birth Defects, Children’s Hospital, Fudan University and Institutes of Biomedical Sciences, Fudan University, Shanghai, 201102 China; 3 Institute of Molecular Embryology and Genetics, Kumamoto University, Kumamoto, Japan; 4 Department of Computational Biology, St. Jude Children’s Research Hospital, Memphis, TN 38105 USA; 5 Hopp Children’s Cancer Center Heidelberg (KiTZ); Division of Pediatric Neurooncology, German Cancer Research Center (DKFZ) and German Cancer Consortium (DKTK), 69120 Heidelberg, Germany; 6 Princess Máxima Center for Pediatric Oncology, Utrecht, the Netherlands; 7 Department of Pediatric Hematology and Oncology, Heidelberg University Hospital, 69120 Heidelberg, Germany; 8 Department of Tumor Cell Biology, St. Jude Children’s Research Hospital, Memphis, TN 38105 USA; 9 Department of Neurology, Pediatrics, and Surgery, Helen Diller Family Comprehensive Cancer Center, University of California San Francisco, San Francisco, CA 94143 USA; 10 Department of Pediatrics, University of Cincinnati, College of Medicine, Cincinnati, OH 45229 USA

**Keywords:** CNS cancer, Cancer genomics, Tumour-suppressor proteins, Cancer genomics

## Abstract

MYC-driven medulloblastomas are highly aggressive childhood brain tumors, however, the molecular and genetic events triggering *MYC* amplification and malignant transformation remain elusive. Here we report that mutations in CTDNEP1, a CTD nuclear-envelope-phosphatase, are the most significantly enriched recurrent alterations in MYC-driven medulloblastomas, and define high-risk subsets with poorer prognosis. *Ctdnep1* ablation promotes the transformation of murine cerebellar progenitors into *Myc*-amplified medulloblastomas, resembling their human counterparts. CTDNEP1 deficiency stabilizes and activates MYC activity by elevating MYC serine-62 phosphorylation, and triggers chromosomal instability to induce p53 loss and *Myc* amplifications. Further, phosphoproteomics reveals that CTDNEP1 post-translationally modulates the activities of key regulators for chromosome segregation and mitotic checkpoint regulators including topoisomerase TOP2A and checkpoint kinase CHEK1. Co-targeting MYC and CHEK1 activities synergistically inhibits CTDNEP1-deficient MYC-amplified tumor growth and prolongs animal survival. Together, our studies demonstrate that CTDNEP1 is a tumor suppressor in highly aggressive MYC-driven medulloblastomas by controlling MYC activity and mitotic fidelity, pointing to a CTDNEP1-dependent targetable therapeutic vulnerability.

## Abstract (web summary)

Group 3 medulloblastomas (MBs) have the worst prognosis amongst the subtypes of MBs and are associated with MYC amplifications. Here the authors identify that mutations in CTDNEP1 cause MYC activation, amplification, and genomic instability in this subtype of MBs.

## Introduction

Medulloblastomas (MBs), which arise from cerebellar neural progenitor cells, are the most common malignant childhood brain tumors, and feature high genomic instability^1^. Based on gene expression and/or DNA methylation profiling MBs are classified into four major subgroups: Wingless (WNT), Sonic Hedgehog (SHH), Group 3 (G3), and Group 4 (G4), with intertumoral heterogeneity within each subgroup^2–4^. Dysregulation of the WNT and hedgehog (HH) pathway has been implicated in WNT and SHH subgroup tumors, respectively, while MYC-driven G3-MB, which comprises ~17% of G3-MBs, has the worst prognosis and is associated with amplification and overexpression of the *c-MYC* oncogene (*MYC*)^1,5–8^. Patients with G3-MB tumors often relapse following therapy, exhibit metastases, and eventually succumb to the disease^3^. Currently, targeted therapeutics for the G3-MB tumors are lacking in part due to the incomplete understanding of tumorigenic mechanisms and clinical correlates of genetic alterations. Although large-scale genomic studies have identified many somatically mutated genes in G3-MB tumors, candidate cancer genes that trigger MYC activation and amplification as well as their underlying regulatory circuitries remain poorly defined.

*MYC* activation or overexpression has been shown to induce genomic instability that is linked to tumor initiation^9–11^ including tumorigenesis in murine G3-MBs from cerebellar progenitor cells^12,13^. Currently, regulatory networks that control MYC activation and transformation of neural precursors into the most aggressive MYC-driven G3 MBs are poorly understood. Recent genomic studies have identified candidate mutations in MB subgroups^3,4,14^, however, no presumptive mutations in the *MYC* gene itself have been found in patients. Recent studies show that MYC protein stabilization and compartmentalization at the nuclear periphery are critical for MYC oncogenic activity, in part mediated through phosphorylation at the critical serine-62 (p62-MYC)^15–17^. In addition, global proteomes and phospho-proteomes indicate that post-translational modifications of MYC such as phosphorylation are associated with poor outcomes in G3 MBs^18^. However, at present, the genetic and molecular pathways that regulate MYC activity or *MYC* amplifications during G3 MB tumorigenesis remain elusive. In this study, by integrating analyses of newly diagnosed MBs and publicly available cohorts for recurrent genetic alterations, we found that *CTDNEP1*, encoding a CTD nuclear envelope-enriched phosphatase (a.k.a *Dullard*)^19–21^, is the most significantly mutated genes within G3-MBs compared with other MB subgroups^4,14^. Mutations or low *CTDNEP1* expression levels define a subset of highly aggressive MYC-driven MBs and predict poor patient outcomes. Notably, ablation of *Ctdnep1* activates MYC oncogenic activity and induces the genomic instability, leading to p53 loss and *Myc* overexpression or amplifications to promote the transformation of cerebellar progenitors into aggressive *Myc*-amplified MBs. We further show that CTDNEP1 modulates activities of key mitotic checkpoint regulators for maintaining genomic stability. Thus, our study provides important evidence showing that mutation in a single gene CTDNEP1 promotes MYC activation and amplifications for G3-MB tumorigenesis, revealing a CTDNEP1 phosphatase-dependent targetable vulnerability in the highly aggressive MYC-driven MB tumors.

## Results

### *CTDNEP1* mutations are most significantly enriched in aggressive G3-MBs and correlated with *MYC* amplification and poor prognosis

**Fig. 1:**
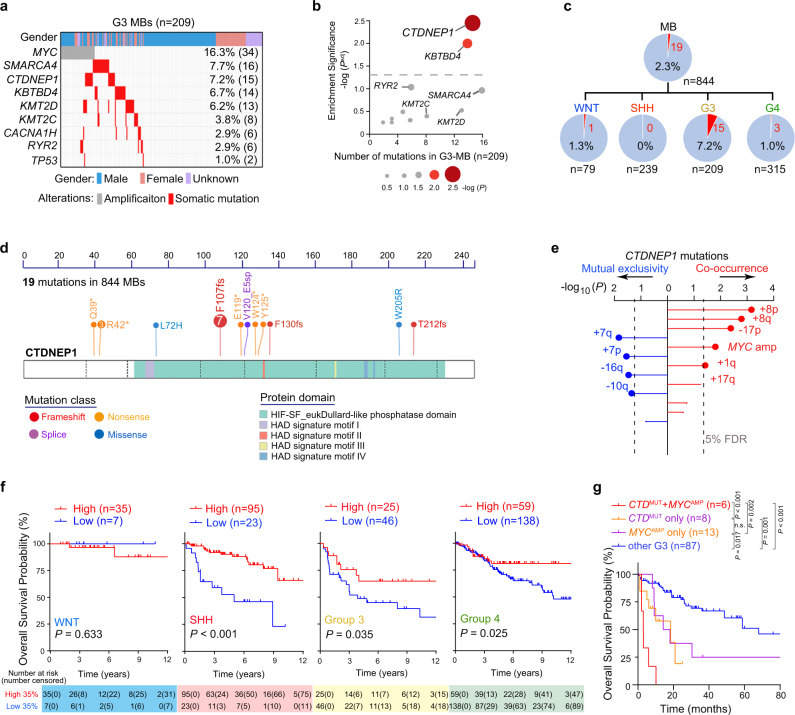
Prevalence and clinical impact of recurrent mutations of CTDNEP1 in G3 MBs. **a** Frequency of known and recurrent genetic variants in pediatric G3 MBs. **b** The significance enrichment plot of somatic recurrent mutated genes in G3-MB compared with other MB subgroups. *P* values were calculated based on Fisher’s exact test and were then adjusted for multiple testing by Bonferroni correction methods. **c** Frequency of *CTDNEP1* LOF variants in different MB subgroups. **d** Somatic *CTDNEP1* mutation profile in patients with MBs. fs, Frameshift. **e** Association between somatic *CTDNEP1* LOF variants and somatic chromosomal alterations (*n* = 136 G3-MB). *p* values were calculated using Bayesian logistic regression analysis, likelihood ratio tests, and adjusted for multiple testing based on 5% false discovery rate (FDR) correction. **f** Kaplan–Meier analysis of overall survival of patients with WNT, SHH, G3, and G4 MB based on the *CTDNEP1* high and low expression across subgroups in publicly available MB cohorts. Log-rank test. **g** Kaplan-Meier analysis of overall survival of patients with *CTDNEP1* (*CTD*) mutation and no *MYC* amplification (*CTD* w/o *MYC*), *MYC* amplification and no *CTDNEP1* mutation (*MYC* w/o *CTD*), *CTDNEP1* mutation and *MYC* amplification (*CTD* + *MYC*) and other G3 MB patients (other G3). Log-rank test. n.s., not significant. Source data are provided as a Source Data file.

To identify the genetic alterations in aggressive MBs, we analyzed whole-exome sequencing data of newly diagnosed MBs cohort from East Asia^22^ and combined with the publicly available MB cohorts^4,5,7,14^ to catalog recurrent somatic mutations. We identified a set of somatic mutations with high frequency among G3 MB samples (*n* = 209), including mutations in *SMARCA4* (16 patients, 7.7%), *CTDNEP1* (15 patients, 7.2%), *KBTBD4* (14 patients, 6.7%), *KMT2D* (13 patients, 6.2%), and *KMT2C* (9 patients, 4.3%) (Fig. 1a), which is in keeping with previous reports^3,4,23^. However, among the recurrent somatic mutations in the publicly available MB cohorts, we found that *CTDNEP1* mutations are most significantly enriched in G3 MBs compared with other MB subgroups (Fig. 1b and Supplementary Fig. 1a). In addition, *CTDNEP1* mutations occur most frequently in MBs compared to other CNS tumor types (Supplementary Fig. 1b), and *CTDNEP1* expression is lower in MB tumors than normal brain and cerebellar tissues (Supplementary Fig. 1c). Among the MBs from the available cohorts, 19 *CTDNEP1* somatic mutations were identified. The majority of patients identified with *CTDNEP1* mutations (15 out of 19) had been diagnosed with G3-MBs, with no or low frequency in patients with WNT (1 out of 19), SHH (0 out 19), or G4 subgroup (3 out of 19) MBs (Fig. 1c). Moreover, *CTDNEP1* mutations were mainly distributed in the critical Dullard-like phosphatase domain or resulted in truncations of this domain (Fig. 1d), suggesting that these mutations may be associated with CTDNEP1 loss-of-function (LOF). We confirmed the somatic *CTDNEP1* mutations in a set of G3 MBs (Supplementary Fig. 2a, b).

We observed loss-of-heterozygosity (LOH) of the other allele of *CTDNEP1* on Chr17p in the majority of the G3 MBs carrying *CTDNEP1* mutations (11 out of 12; Supplementary Fig. 2c–f), while LOH was not detected in the normal apparent peri-tumoral tissue in the same patient (Supplementary Fig. 2c). In contrast, in G4 MBs, which do not have high MYC levels^24^, *CTDNEP1* mutations were detected in only three G4 MBs (3 out of 173) with one tumor showing i17q (Supplementary Fig. 2g). The *CTDNEP1* expression level was lower in Chr17p-deleted MBs compared to those with balanced Chr17p in G3 or G4 MBs and SHH or WNT MBs (Supplementary Fig. 3a–c).

To examine the relation of *MYC* amplification and *CTDNEP1* mutations to genomic alterations in G3 MB tumors, we analyzed the copy number variation from the available cohorts^3,14,22,24^. In G3 MBs, we found that *CTDNEP1* mutations significantly co-occurred with *MYC* amplification, copy number gains on Chr8p, Chr8q, Chr17q, and Chr1q, as well as the loss of Chr17p, while the mutually exclusive events included gain of Chr7p or Chr7p and loss of Chr16q or Chr10q (Fig. 1e and Supplementary Fig. 2c, d). The co-occurrence of isochromosome 17q (i17q) and Chr8q gain was observed in both *CTDNEP1*-mutated and *MYC*-amplified MBs (Fig. 1e and Supplementary Fig. 2e, f). Together, these observations indicate that *CTDNEP1* mutation is correlated with *MYC* amplification and genomic instability in G3-MB tumors.

Due to variable expression levels, we stratified the MB cohorts from publicly available datasets^3,4,23^ into patient populations with high and low *CTDNEP1* expression across MB subgroups (Supplementary Fig. 3d), and found that lower *CTDNEP1* expression level was correlated with significantly decreased overall survival in SHH-, G3- and G4-MB cohorts (Fig. 1f and Supplementary Fig. 3e), except for the WNT subgroup, although few *CTDNEP1* mutations were present in SHH and G4 MBs. Based on the survival data^3,4,23^, patients carrying *CTDNEP1* somatic mutations exhibited a worse prognosis than those with *CTDNEP1* wildtype alleles in G3 MB tumors without *MYC*-amplification, but similar to those with MYC-amplification (Fig. 1g). Among G3 MB tumors, the patients with both *CTDNEP1* mutation and *MYC* amplification showed the poorest prognosis when compared with those with the *CTDNEP1* mutation or *MYC* amplification alone (Fig. 1g). Together, these observations suggest that the prevalence and clinical impact resulting from *CTDNEP1* mutations or low expression define a subset of highly aggressive G3-MBs.

### CTDNEP1 deficiency promotes MB tumor cell growth

**Fig. 2:**
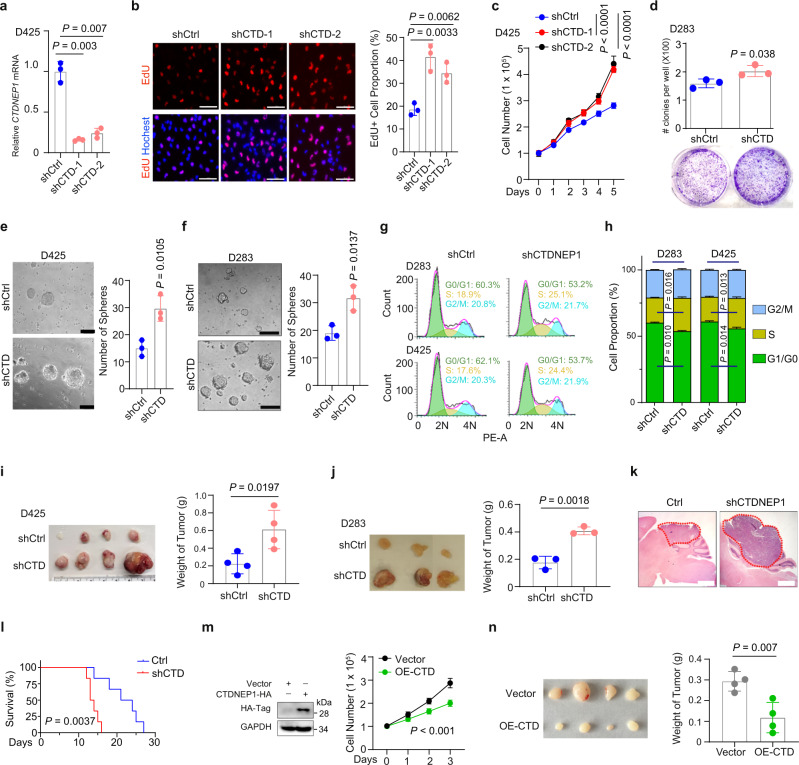
Inhibition of *CTDNEP1* expression promotes MB cell proliferation. **a** qRT-PCR quantification of *CTDNEP1* in D425 cells transduced with lentiviral control (shCtrl) and shRNAs targeted against *CTDNEP1* (shCTD). *n* = 3 independent experiments. **b** Left, representative images of shCtrl and shCTD-transduced D425 cells stained for EdU (scale bar, 50 μm); right, quantification of EdU^+^ cells. *n* = 3 independent experiments. **c** Growth of shCtrl and shCTD-transduced D425 cells assayed by WST-1. *n* = 6 independent measurements. Two-way ANOVA. **d** Upper, the number of clones per well in a soft agar assay of Ctrl and shCTD D283 cells after 10 days culture. Lower: representative images of clones in soft agar plates. *n* = 3 independent experiments. **e**, **f** Left: representative images of neurospheres of control and shCTD- transduced D425 (**e**) and D283 cells (**f**). Scale bars, 100 μm. Right panels: the number of spheres. *n* = 3 independent experiments. **g**, **h** Representative flow cytometry (**g**) of cell-cycle stages of D283 (upper) and D425 (lower) cells transduced with shCtrl or shCTD. **h** percentage of cells at different cell-cycle stages (*n* = 3 independent experiments), ns: no significance. **i**, **j** Left: representative photographs of tumors from mice transplanted subcutaneously with 1 × 10^6^ shCtrl and shCTD-treated D425 (**i**), or D283 (**j**) cells. Right: weights of tumors as means ± SD (*n* = 4 mice per group for **i** and *n* = 3 mice for **j**). **k**, **l** Representative hematoxylin/eosin staining of the cerebellum from the mice transplanted with shCtrl and shCTDNEP1-transduced MB-004 cells (**k**) and their survival curves (**l**; *n* = 6 animals/group). Log-rank test. Dash circles indicate the tumor tissues. Scale bars: 1 mm. **m** Left: Immunoblots of CTDNEP1 overexpression (OE) in D425 cells. Right: cell proliferation as monitored by WST-1 in control and CTD-OE D425 cells. *n* = 3 independent experiments, two-way ANOVA. **n** Left: photographs of tumors from mice transplanted subcutaneously with control or CTDNEP1-overexpressing D425 cells. Right: Weights of tumors (*n* = 4 animals per group). The data are presented as mean values ± SD. Two-tailed Student’s *t* test for **a**, **b**, **d**–**f**, **h**–**j**, **n**. Source data are provided as a Source Data file.

To determine the effect of CTDNEP1 deficiency on tumor cell growth, we inhibited *CTDNEP1* expression in different G3-MB tumor cells with or without *MYC* amplification using lentiviral shRNAs. In *MYC*-amplified human G3-MB cell lines D425 and MB-004 (Supplementary Fig. 4a)^25^, *CTDNEP1* knockdown resulted in an increase in cell proliferation measured as EdU or BrdU incorporation assays (Fig. 2a, b and Supplementary Fig. 4b) and cell growth rates (Fig. 2c and Supplementary Fig. 4c). In addition, by using a soft agar assay, we found that silencing of *CTDNEP1* resulted in a higher clonogenic capacity in non-*MYC* amplified G3 MB cells (D283)^25^ (Fig. 2d) and MYC amplified D425 cells^25^ (Supplementary Fig. 4d). Moreover, *CTDNEP1* knockdown led to significant increases in tumor sphere formation in both *MYC* amplified (D425) and non-*MYC* amplified (D283) G3-MB cell lines (Fig. 2e, f). Cell-cycle analysis using flow cytometry revealed that CTDNEP1 deficiency also increased the proportion of these G3 MB cells in S phase (Fig. 2g, h). These results indicate that CTDNEP1 deficiency promotes the proliferation of G3-MB cells in vitro.

To assess the in vivo effect of *CTDNEP1* inhibition on tumor formation, D425 or D283 MB cells with or without *CTDNEP1* knockdown were subcutaneously transplanted into NOD SCID gamma (NSG) mice. The sizes of tumors derived from *CTDNEP1*-deficient cells were much larger than those from control shRNA-treated cells (Fig. 2i, j). In addition, knockdown of CTDNEP1 in patient-derived MB-004 cells accelerated tumor growth and shortened animal lifespan in an orthotopic-engrafted model (Fig. 2k, l). Thus, the loss of CTDNEP1 promoted tumor growth of both *MYC-*amplified and non-*MYC-*amplified G3-MB cells in xenografts, suggesting a tumor-suppressive role for CTDNEP1 in G3-MBs.

To investigate the effect of CTDNEP1 overexpression on tumor cell growth, we transduced D425 MB cells with a lentivirus overexpressing CTDNEP1. CTDNEP1-overexpression resulted in a reduction of the growth rate of D425 cells in vitro (Fig. 2m). In addition, tumors in mice xenografted with CTDNEP1-overexpressing D425 cells were smaller than those grafted with control vector-transduced D425 cells (Fig. 2n), suggesting that CTDNEP1 overexpression inhibits tumor cell growth. Together, these observations suggest that CTDNEP1 has a tumorgrowth suppressive activity in G3-MB cells.

### CTDNEP1 depletion leads to activation of MYC signaling pathway

**Fig. 3:**
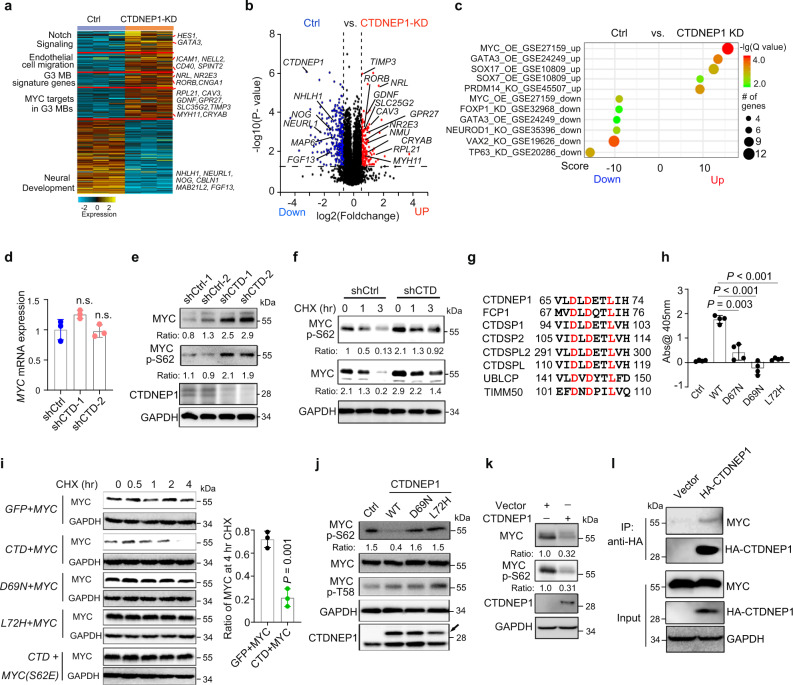
Loss of CTDNEP1 increases MYC stability and activity. **a** Heatmap of differentially expressed genes in D425 cells treated with shCtrl and shCTD. **b** Volcano plot of transcriptome profiles between control D425 cells and shCTDNEP1 (CTDNEP1-KD) treated cells. Red and blue dots represent genes significantly upregulated and downregulated in cells depleted of CTDNEP1 (*p* < 0.05, false discovery rate [FDR] < 0.1, two-sided Student’s *t* test for correction for multiple hypotheses testing), respectively. **c** Correlations of upregulated genes in shCTDNEP1 D425 cells with MYC expression in G3 MBs. Benjamini-Hochberg method with correction for multiple hypothesis testing of significance. **d** qRT-PCR quantification of *c-MYC* mRNA in shCtrl and shCTD-treated D425 cells. n.s., not significant. **e** Representative immunoblots from 2 independent experiments for total MYC protein and p-S62-MYC in shCtrl and shCTD-treated D425 cells. **f** Representative immunoblots from 3 independent experiments for total MYC protein and p-S62-MYC in shCtrl and shCTD D425 cells treated with cycloheximide (CHX, 10 μg/ml) for 1 or 3 h. **g** Conserved residues (red) in the catalytic motif of DXDX(T/V) protein family. **h** The phosphatase activity of CTDNEP1 wildtype and mutant (D67N, D69N, and L72H) proteins. **i** Representative immunoblots from 3 independent experiments for flag-tagged c-MYC after treatment by CHX at indicated time points in 293 T cells co-transfected with wildtype (wt) or flag-tagged c-Myc mutant (S62E), and wildtype (wt) or myc-tagged CTDNEP1 mutants (D69N or L72H) as indicated (left). Quantification of c-Myc-flag expression after 4 hr CHX treatment as compared to untreated cells (right). **j** Representative immunoblots for MYC and p-Ser62 MYC levels in D425 cell lysates after 1 h incubation with wild-type and mutants of HA-tagged CTDNEP1 proteins. **k** Representative immunoblots for MYC protein and p-S62-MYC in stable CTDNEP1 overexpressing D425 cells. **l** Representative immunoblots for CTDNEP1 with MYC in D425 cells transduced with control and lentivirus expressing HA-tag-CTDNEP1 after 4-h of MG132 treatment. *n* = 3 independent experiments in **a**, **e**, **f**, **h**–**l**. The data are presented as mean values ± SD. Two-tailed Student’s *t* test for **d**, **h**, and **i**. Source data are provided as a Source Data file.

To further determine the potential mechanisms underlying the tumor suppressive effects of CTDNEP1, we performed transcriptome profiling of D425 MB cells transduced with control shRNAs and sh*CTDNEP1* RNAs. We identified a set of genes that were significantly altered (>1.5-fold change, *p* < 0.01) in *CTDNEP1*-depleted cells (Fig. 3a, b). Among the most upregulated genes were those pertinent to tumor progression, including the signature genes for G3 MBs (e.g., *NRL*, *NR2E3*, and *RORB*)^3,26^ and candidate MYC-targeted genes (e.g., *RPL21*, *CAV3*, *GDNF, GPR27, TIMP3,* and *SLC35G2*)^27^ (Fig. 3a, b). In addition, the genes associated with NOTCH signaling (e.g., *HES1*, *GATA3*, and *MAGEA1*) and cell migration (e.g., *ICAM1*, *NELL2*, *CD40*, *SLIT2*, and *SPINT2*) were also upregulated upon *CTDNEP1*-knockdown (Fig. 3a, b). In contrast, the downregulated genes were associated with normal neural development (Fig. 3a). Markedly, gene set enrichment analysis (GSEA)^28^ indicated that the upregulated genes showed strong enrichment in transcriptomic profiles of MYC-pathway target genes (Fig. 3c). These observations suggest that depletion of *CTDNEP1* results in MYC signaling activation. Consistently, the analysis of human G3-MB transcriptomic profiles^4^ revealed that *CTDNEP1-*mutated MBs exhibited a similar expression pattern with MYC-high or -amplified MBs (Supplementary Fig. 4e). Together, these observations suggest that *CTDNEP1* deficiency leads to MYC upregulation or activation of the MYC oncogenic pathway.

### CTDNEP1 phosphatase activity loss leads to MYC stabilization and phosphorylation at Ser62

Despite the increase in MYC target expression in *CTDNEP1*-deficient cells, *MYC* mRNA levels were not substantially altered in D425 tumor cells treated with sh*CTDNEP1* RNAs (Fig. 3d), suggesting that CTDNEP1 might regulate MYC signaling post-translationally. Western blotting analysis indicated that there was an increase of protein levels in MYC and pS62-MYC, which leads to MYC stabilization and functional activation^29^, in the *CTDNEP1-*knockdown cells (Fig. 3e). Given that *CTDNEP1* encodes a nuclear envelope-enriched serine/threonine protein phosphatase^19,30^ (Supplementary Fig. 5a), we hypothesized that CTDNEP1 activity destabilizes MYC via removal of the phosphate from S62, which is critical for MYC oncogenic activity at the nuclear periphery^15–17^. To examine MYC stability, we treated tumor cells with a protein synthesis inhibitor cycloheximide (CHX) and found that both MYC and p-S62-MYC levels remained higher in the *MYC*-amplified D425 and non-*MYC* amplified D283 cells with *CTDNEP1* knockdown than control-treated cells (Fig. 3f and Supplementary Fig. 5b, c). These data suggest that *CTDNEP1* depletion leads to an increase in MYC stability and oncogenic activity.

To determine whether dephosphorylation of MYC at S62 is catalyzed by CTDNEP1, we generated constructs carrying a mutation at the codon D67 (D67N) or D69 (D69N) required for CTDNEP1 phosphatase activity^19^. Furthermore, we constructed an expression vector carrying a G3-MB patient-derived CTDNEP1 mutation L72H to examine the impact of the disease-relevant mutation on CTDNEP1 activity. Three residues (D67, D69, and L72) are highly conserved in the catalytic motif DXDX(T/V) among the phosphatase protein family (Fig. 3g). The phosphatase activity of affinity-purified wild-type and CTDNEP1 mutant proteins was assessed using p-nitrophenyl phosphate as a substrate^19^. The wild-type enzyme catalyzed dephosphorylation of p-nitrophenyl phosphate, but none of the CTDNEP1 mutants did (Fig. 3h), suggesting that the CTDNEP1 mutants are defective in phosphatase activity.

We next co-expressed CTDNEP1 or its activity-deficient mutants along with MYC, and found that overexpression of CTDNEP1, but not the mutants, increased MYC degradation (Fig. 3i). Importantly, in further support of its potential role in regulating MYC levels by dephosphorylating it at S62, CTDNEP1 overexpression did not alter the stability of the non-phosphorylatable MYC-S62E mutant (Fig. 3i). To confirm that CTDNEP1 dephosphorylates MYC, CTDNEP1 and its mutants D69N and L72H were incubated with the lysates of D425 cells, which express a high level of MYC. p-S62-MYC levels were substantially reduced in the presence of wildtype CTDNEP1 but were not altered in the presence of these CTDNEP1 mutants (Fig. 3j). Consistently, overexpression of *CTDNEP1* in D425 cells substantially downregulated the levels of MYC and p-S62-MYC (Fig. 3k). In addition, co-immunoprecipitation assays indicated that CTDNEP1 was associated with MYC in a complex in D425 G3 cells transduced with the lentivirus expressing *CTDNEP1* (Fig. 3l). These observations indicate that CTDNEP1 may interact with and dephosphorylate MYC at S62 to regulate MYC stability.

### CTDNEP1 phosphatase activity regulates MYC expression in nucleus and nuclear membrane

To investigate the dynamics of nucleoplasmic and nuclear membrane-associated MYC in relation to the phosphatase activity of CTDNEP1, we found that overexpression of wildtype CTDNEP1 led to a decrease in MYC levels in the nucleus (Supplementary Fig. 6a, b) and p-S62 MYC levels in both nucleoplasmic and nuclear membrane (Supplementary Fig. 6c, d). In contrast, expression of the phosphatase-activity-deficient CTDNEP1-D69N did not substantially affect the expression levels of MYC and p-S62 MYC in the nucleoplasm (Supplementary Fig. 6a–d).

To determine whether CTDNEP1 can directly interact and co-localize with MYC and p-S62 MYC at the nuclear periphery, we performed the proximity ligation assay (PLA) in HeLa cells, which have been used as a cell system to examine the MYC expression or p62-MYC localization^16^. Expression of HA-tagged CTDNEP1 was mainly localized around the nuclear envelope/periphery (Supplementary Fig. 6a–d), consistent with enrichment of CTDNEP1 at the nuclear envelope as previously reported^19^. PLA assays indicated co-localization of CTDNEP1 with MYC and p-S62 MYC at the nuclear periphery (Supplementary Fig. 6e, f). Moreover, we detected an increase in the co-localization of phosphatase-activity-deficient CTDNEP1-D69N with MYC and p-S62-MYC on the nuclear periphery compared with wildtype CTDNEP1 (Supplementary Fig. 6e, f), consistent with our observation that the defects in CTDNEP1 phosphatase activity resulted in an increased MYC S62 phosphorylation and stabilization. Together, these data suggest that CTDNEP1 activity can be executed at least in part at the nuclear periphery and regulates the expression levels of nucleoplasmic and nuclear membrane-associated MYC protein and p-S62 MYC.

### Deletion of *Ctdnep1* induces neural progenitor transformation into MYC-driven G3 MB tumors

**Fig. 4:**
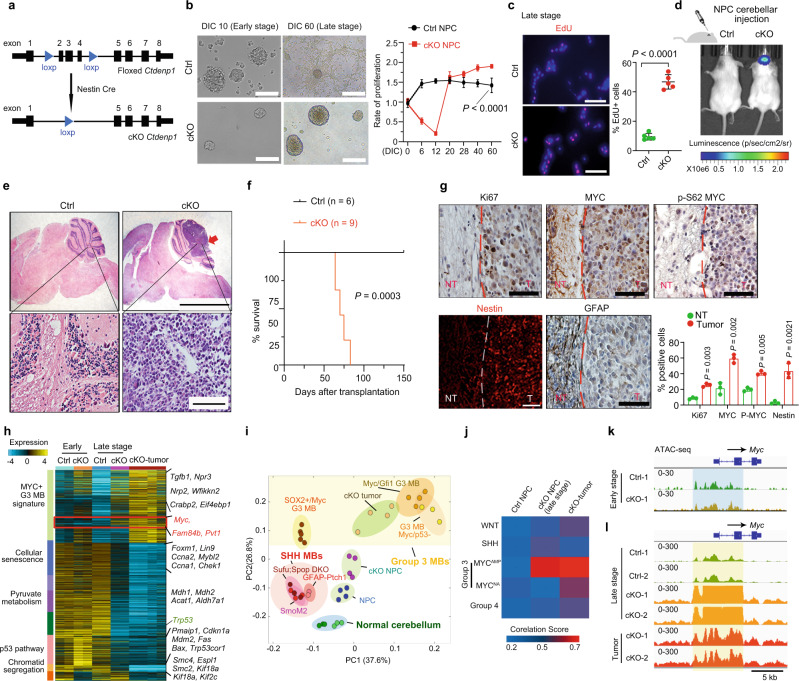
*Ctdnep1* ablation in cerebellar NPCs induces G3 MB-like tumor formation. **a** Diagram depicting the generation of *Ctdnep1-*cKO mice. **b** Left and middle, representative images (left) of *Ctdnep1-*cKO NPCs (cKO) and control (Ctrl) NPCs at different stages. Right, relative proliferative rates of control and *Ctdnep1*-cKO NPCs at the different stages of in vitro culture (right). *n* = 5 independent experiments. Scale bars: 100 μm. **c** Left: Representative images of EdU stained control and *Ctdnep1-*cKO NPCs. Scale bar: 100 μm. Right: Percentages of EdU+ cells (*n* = 5 independent experiments) in *Ctdnep1-*cKO or WT NPC at late-stage (DIC 60). **d** Representative bioluminescence imaging of mice transplanted with WT or *Ctdnep1-*cKO NPCs at 65 days post-transplantation. **e** Hematoxylin and eosin staining of cerebellum sections with WT or *Ctdnep1-*cKO NPCs transplantation. Scale bars: 5 mm (upper) and 100 μm (lower). *n* = 5 independent allografts/group. **f** Survival curves for animals transplanted with Ctrl and *Ctdnep1-*cKO NPCs. Log-rank test. **g** Representative immunostaining and quantification of the labeled cells in tumor and para-tumor regions from *Ctdnep1-*cKO NPCs transplanted cerebella. Scale bars: 50 μm. *n* = 3 independent allografts/group; NT, non-tumor region of the samples with tumor; T, tumor tissue. **h** Heatmap of differentially expressed genes in *Ctdnep1-*cKO tumors, *Ctdnep1-cKO* NPCs, and Ctrl NPCs. *n* = 2 independent samples/group. **i** Principal component analysis of transcriptomes of *Ctdnep1-*cKO NPCs (DIC 60) and cKO tumors with mouse SHH (SmoM2 OE^36^ and *GFAP*-cKO *Ptch1*^91^) and G3 MB (Myc_Gfi1 tumor^92^, Myc/Trp53- Group3 MB^93^, and Sox2+ Myc^35^ models and normal cerebella^36,37^. **j** Correlation of transcriptomic profiles of *Ctdnep1*-cKO NPCs (DIC 60) and cKO tumors with human MB subgroups using MB signature genes. MYC^AMP^ and MYC^NA^ represent MYC amplification and non-amplification G3-MBs, respectively. **k**, **l** Genome browser tracks of ATAC-seq signals at the locus of *Myc* in control NPCs and *Ctdnep1*-cKO NPCs at DIC 10 (**k**) or DIC 60 or *Ctdnep1*-cKO tumor cells (**l**). Highlights: the promoter/enhancer of *Myc*. The data are presented as mean values ± SD. Two-tailed Student’s *t* test for **b**, **c**, **g**. Source data are provided as a Source Data file.

To gain insight into the role of CTDNEP1 in MB tumorigenesis in vivo, we knocked out *Ctdnep1* in neural stem/progenitor cells (NPCs) in mice by breeding *Ctdnep1*^flox/flox^ mice^20^ with a Nestin-Cre line^31^ to generate *Ctdnep1*^flox/flox^; Nestin-Cre mice referred to here as *Ctdnep1-*cKO mice (Fig. 4a). Strikingly, all *Ctdnep1*-cKO mice died before postnatal day 40 (Supplementary Fig. 7a), and the cortex and cerebellum of the animals lacking *Ctdnep1* were significantly smaller compared with controls (Supplementary Fig. 7b, c). We observed an increase of DNA damage responses (marked by γH2AX) and apoptosis (marked by cleaved caspase 3) in the cerebellar progenitor cells during embryonic development in *Ctdnep1*-cKO animals (Supplementary Fig. 7d), suggesting that *Ctdnep1* deletion results in DNA damage and cell death in a population of cerebellar progenitors during development.

The smaller size and increase of cell death in the cerebellum of *Ctdnep1*-deficient animals suggests that CTDNEP1 may control cerebellar NPC development. To determine the effect of *Ctdnep1* deficiency on NPC growth, we isolated cerebellar NPCs from control and *Ctdnep1-*cKO mice on postnatal day 4. *Ctdnep1-*cKO NPC spheres appeared smaller than wildtype NPCs during early stages in culture, which is potentially due to apoptosis in a population of mutant cells, however, the growth of *Ctdnep1-*cKO NPCs rapidly accelerated and exhibited substantially higher proliferation rate than control NPCs at late stages e.g., at 60 days in culture (DIC 60) compared with the early stage DIC 10 (Fig. 4b, c). This suggests that a population of *Ctdnep1-*deficient NPCs acquired a growth advantage at the later stages.

To evaluate the capacity of *Ctdnep1-*cKO NPCs to cause tumorigenesis, we orthotopically transplanted the luciferase-expressing NPCs from control and *Ctdnep1-*cKO mice into the cerebella of NSG mice. No tumors formed in mice transplanted with control NPCs, but the animals transplanted with *Ctdnep1*-cKO NPCs at DIC 60 developed tumors in the cerebellum with full penetrance (14 of 14 mice), as detected by bioluminescence analyses (Fig. 4d). The tumors had a large cell/anaplastic (LC/A) morphology (Fig. 4e) and resemble that observed in human MYC-driven G3 MB^32^. The animals transplanted with *Ctdnep1-*cKO NPCs had a short lifespan and died around 90 days post-transplantation (Fig. 4f). To evaluate the tumorigenicity of *Ctdnep1*-deficient neoplastic cells, we transplanted primary neoplastic cells from allografts at varying cell doses into secondary recipients orthotopically and generated tumors with full penetrance (Supplementary Fig. 8a, b), suggesting that *Ctdnep1*-deficient neoplastic cells are tumorigenic and enable aggressive MB formation.

Immunohistochemical characterization indicated that cells in the tumors derived from *Ctdnep1-*cKO NPCs had a significantly higher proliferative rate than normal cerebellar regions, as assayed by Ki67 (Fig. 4g). In addition, these tumors exhibited strong expression of the oncogenic factor MYC and p-S62 MYC as well as stem cell/progenitor marker Nestin, but weak staining for the astrocytic marker GFAP (Fig. 4g), which are characteristics similar to previously described G3-MB mouse models^12,13^. Notably, *Ctdnep1*-deficient NPCs transplanting from early-stage culture (e.g., DIC 15) into nude mice propagated into the same type of tumors with MYC overexpression (Supplementary Fig. 8c, d). Together, these results suggest that a population of NPCs acquires the tumorigenic potential in the absence of *Ctdnep1*.

Since the loss-of-function p53 cooperates with MYC overexpression to promote the formation of G3-like MB^12,13^, we then transduced freshly isolated NPCs from *Ctdnep1*-cKO animals with retroviruses expressing dominant-negative p53 (DNp53). The *Ctdnep1*-cKO NPCs transduced with DNp53 after 24 hr were transplanted into the cerebellum of NSG mice and were able to form G3 MB-like tumors (6 out 8) in allografts orthotopically with upregulation of MYC and pS62 - MYC expression and Ki67+ proliferative cells (Supplementary Fig. 8e–h), while DNp53-transduced wildtype NPCs did not form tumors, suggesting that p53 loss-of-function enhances the tumorigenic potential of *Ctdnep1*-deficient NPCs.

Transcriptomic analysis indicated that *Ctdnep1*-cKO tumor cells had higher levels of G3-MB signature genes (e.g., c-*Myc* and *Npr3*)^33^ than NPCs from normal cerebella (Fig. 4h). Principal component analysis showed that the gene profiles of *Ctdnep1*-cKO tumor cells had a closer relationship to those of murine MYC-driven G3-MB tumors from NPCs (e.g. Myc/Trp53^−/−^ and Myc_Gfi1 MB models)^12,13,34^ than those from astrocyte progenitors (Sox2^+^ Myc model)^35^, SHH-MB tumors (e.g. SmoM2 OE and *Ptch1* models)^36,37^ or normal cerebella (Fig. 4i). In addition, the expression profile of *Ctdnep1*-cKO-tumor cells exhibited a greater similarity to that of MYC-driven G3 MB mouse models when compared with *Ctdnep1*-cKO NPCs and control NPCs (Fig. 4i). To further define the subgroup of *Ctdnep1*-cKO tumors, we compared the signature genes of human MB subgroups^3^ to those of *Ctdnep1*-cKO tumors. The *Ctdnep1*-cKO tumors more closely resembled the human MYC-amplified G3-MB than non-MYC-amplified G3-MBs and other human MB subgroups^4,32^ (Fig. 4j). *Ctdnep1*-cKO-tumors exhibited a higher correlation score than *Ctdnep1*-cKO NPCs when compared with human group 3 MB (Fig. 4j). Together, these observations indicate that *Ctdnep1*-loss-induced mouse MBs resemble the aggressive human MYC-driven MBs.

To further investigate how loss of *Ctdnep1* drives gene expression profiles that underlie the development of aggressive MBs, we examined the genomic landscape and chromatin accessibility by performing ATAC-seq (Assay for Transposase-Accessible Chromatin using sequencing)^38^ and observed alterations in accessible chromatin sites in the absence of *Ctdnep1* (Supplementary Fig. 9a). At the early stage DIC 10, chromatin accessibility at the *Myc* locus in *Ctdnep1*-cKO NPCs was comparable to wildtype NPCs (Fig. 4k). However, we observed strong ATAC-seq peak signals in the regulatory regions of the *MYC* locus in *Ctdnep1*-cKO NPCs at the late stage DIC 60 and *Ctdnep1*-cKO tumors compared to control NPCs (Fig. 4l). This suggests an increase of open chromatin accessibility for MYC expression in *Ctdnep1*-deficienct cells at the late tumorigenic stage but not at the early stage. In addition, gene loci associated with G3-MB (e.g., *Kcnj2*, *Ccnd1*, and *Tgfb3*) were more accessible in the *Ctdnep1*-cKO NPCs, whereas the accessibility of genes that regulate normal chromosome segregation (e.g., *Kif2c*, *Kif18a*, and *Espl1*) was reduced (Supplementary Fig. 9b, c). These data suggest that *Ctdnep1* loss promotes tumorigenic programs at least partially through the activation of MYC-driven G3 oncogenic pathways.

### Sustained *Ctdnep1* deletion results in p53 downregulation and MYC upregulation during malignant transformation

**Fig. 5:**
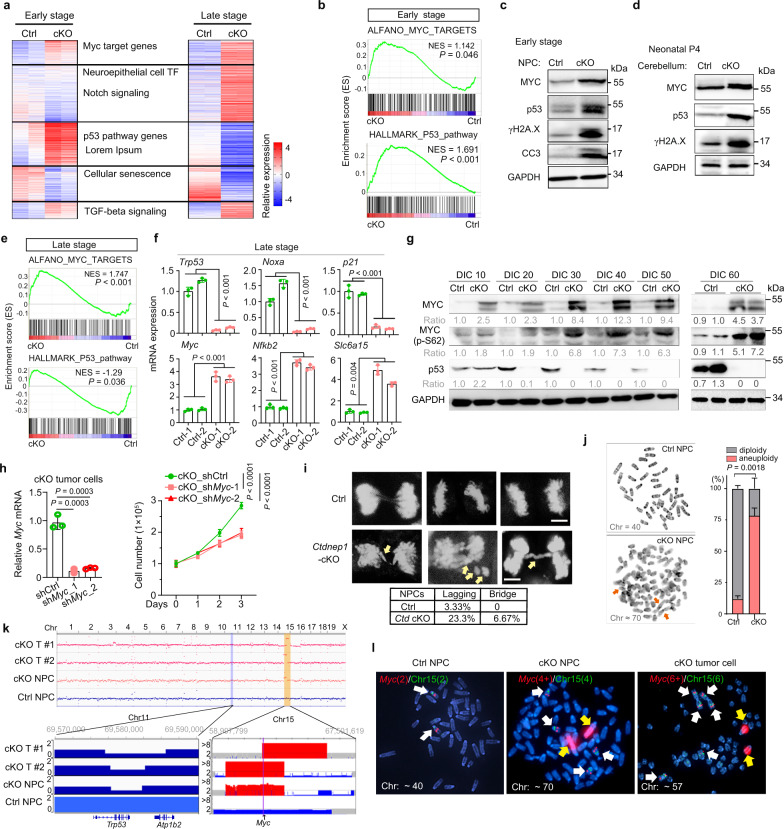
*Ctdnep1* ablation induces genomic instability and *Myc* gene amplification. **a** Heatmap of differentially expressed genes in *Ctdnep1-*cKO NPCs (*n* = 2) compared to wild-type NPCs (Ctrl, *n* = 2). **b** GSEA plots of p53 pathway and MYC target genes between control and *Ctdnep1*-cKO NPCs at the early stage. **c**, **d** Representative immunoblots of indicated proteins in the early-stage NPCs (**c**) and the cerebellum of *Ctdnep1*-cKO mice at postnatal day 4 (**d**). **e** GSEA plots of p53 pathway and MYC target genes between control and *Ctdnep1-*cKO NPCs at the late-stage. **f** qRT-PCR quantification of indicated transcripts in control and *Ctdnep1*-cKO NPCs at late-stage. **g** Representative immunoblots for MYC and p53 in *Ctdnep1-*cKO and Ctrl NPCs from the different stages. **h** Relative *Myc* expression (left) in *Ctdnep1*-cKO NPCs transduced with control shRNA or sh*Myc* RNAs. Cell viability (right) of Ctdnep1-cKO NPCs is measured by WST-1 assay. Right: two-way ANOVA. **i** Anaphase analysis of control and *Ctdnep1-*cKO NPCs at early stage. Upper: representative images of anaphase cells. Scale bar, 5 μm. Lower: Quantification of cells with lagging or bridged chromosomes (*n* = 60 anaphase cells each group). **j** Representative images of karyotype analysis (left) and quantification (right) of control and *Ctdnep1*-cKO NPCs at DIC 70. Arrows: chromosomal aberrations. **k** Upper: CNV analysis of Ctrl NPCs and *Ctdnep1-*cKO NPCs at late-stage (DIC 60), and two independent *Ctdnep1-*cKO tumor cells (cKO-T) based on 30x WGS analysis. Lower: Red and blue regions represent the focal amplified or deleted segments of the indicated chromosome, respectively. **l** Representative FISH images showed the *Myc* gene (Red) and chromosome 15 (Green) in control NPCs at DIC 70 (16/20), *Ctdnep1-*cKO NPCs (cKO-NPC) at DIC 70 (17/20), and *Ctdnep1-*cKO tumor cells (14/20). White arrows, the *Myc* gene in chromosome 15; yellow arrows, *Myc* gene amplification translocated out of chromosome 15. *n* = 3 independent experiments in **c**, **d**, **f**–**h**, **j**. The data are presented as mean values ± SD. GSEA tests in b and e use a standard *t* test statistic. Two-tailed Student’s *t* test for **f**, **h**, **j**. Source data are provided as a Source Data file.

Analysis of transcriptome profiling revealed that the expression levels of both MYC and p53 pathway genes were upregulated in *Ctdnep1*-cKO NPCs at early stage (DIC 12) compared to wild-type NPCs (Fig. 5a, b). MYC upregulation alone has been shown to induce DNA damage, activation of DNA damage responses, and cell death^39,40^. Consistent with this, we observed an upregulation of DNA damage-related markers, γH2A.X and p53, in *Ctdnep1-*cKO NPCs at the early stage and in developing cerebella in mice (Fig. 5c, d). Similarly, acute *Ctdnep1* ablation in NPCs resulted in an activation of a DNA damage response marker γH2A.X and followed by an upregulation of cell death assayed by cleaved-caspase 3 (Supplementary Fig. 10), suggesting that acute *Ctdnep1* deletion induces DNA damage and cell apoptosis.

When examining *Ctdnep1*-cKO NPC tumorigenic cells at the late stage (DIC 60), we found that, despite upregulation of MYC targets, the p53 pathway was downregulated compared with wild-type cerebellar NPCs (Fig. 5e). qRT-PCR analysis confirmed the downregulation of p53 pathway genes (e.g., *Trp53, Noxa*, and *p21*)^41^ and upregulation of MYC pathway genes (e.g., *c-Myc, Nfkb2*, and *Slc6a15*)^42^ in *Ctdnep1*-cKO NPCs at DIC 60 (Fig. 5f). Consistently, western blot analysis indicated an upregulation of MYC and p-S62 MYC, while p53 was progressively downregulated in the *Ctdnep1-*cKO NPCs over the course of tumorigenic transition in culture (Fig. 5g).

To determine if MYC is essential for hyperproliferative growth in the *Ctdnep1-*cKO tumor cells, we knocked down *Myc* utilizing a lentiviral shRNA and found that depletion of *c-Myc* strongly reduced the growth of *Ctdnep1*-cKO tumor cells (Fig. 5h), suggesting that tumor cell growth mediated by *Ctdnep1*-deficiency is dependent upon MYC levels. Together, these observations indicate that sustained *Ctdnep1* deficiency induces the activation of MYC signaling, which can further downregulate the p53 tumor suppressor^43^, leading to malignant transformation of neural progenitors.

### Loss of *Ctdnep1* induces p53 loss and *Myc* amplification

MYC upregulation together with p53 loss has been shown to induce chromosome instability and enables cell survival with DNA damage^40,44–46^. Our transcriptome profiling analysis indicated a downregulation of mitotic sister-chromatid segregation pathway in *Ctdnep1*-cKO tumor cells (Fig. 4h). We then analyzed chromosome segregation during mitosis via DAPI staining in *Ctdnep1-*ablated and wild-type NPCs and observed a substantial increase in chromosome segregation defects such as lagging or bridging chromosomes in *Ctdnep1-*ablated NPCs compared to control NPCs (Fig. 5i). Consistently, karyotype analysis showed that the majority of the *Ctdnep1*-ablated NPCs evaluated at DIC 45 (approximately 55%) exhibited aneuploidy, including triplication of chromosome 15 carrying the *Myc* gene (Supplementary Fig. 11a). In addition, the frequency of chromosomal aneuploidy increased in *Ctdnep1*-ablated NPCs at the late stage e.g., DIC 70 (Fig. 5j). The observations suggest that *Ctdnep1* depletion leads to chromosome mis-segregation and aneuploidy, which is generally associated with a poorer prognosis^47,48^, and may drive the aggressive nature of *Ctdnep1*-deficient MBs.

To further confirm the *Myc*-locus-specific amplification, we performed whole genome sequencing (WGS) analysis and found that the *Myc* gene locus was amplified in late-stage *Ctdnep1*-cKO NPC and *Ctdnep1-*cKO-tumor cells, along with *Trp53* gene loss-of-heterozygosity (Fig. 5k). Notably, the fluorescent in situ hybridization (FISH) assays detected six and four *Myc* amplicons in the majority of *Ctdnep1*-cKO tumor cells (17/20) and *Ctdnep1*-cKO NPCs (14/20) at DIC 70, respectively, compared with the normal two *Myc* gene copies in control NPCs (Fig. 5l). The strongest *Myc* amplicon signals at metaphase were detected in two rearranged chromosomes in *Ctdnep1-*cKO NPCs and *Ctdnep1-*cKO tumor cells (Fig. 5l, yellow arrows). These data indicate that *Ctdnep1* deletion leads to *Myc* amplifications and chromosomal aneuploidy at least in part through focal copy number gain, consistent with elevated *MYC* amplicons in human G3 MB^49^. Consistent with MYC-induced deregulation of cell mitosis^50^, we found that MYC overexpression alone increased anaphase chromosome mis-segregation in D283 cells but did not alter CTDNEP1 levels (Supplementary Fig. 11b–d), indicating that MYC elevation results in aberrant mitotic phenotypes in MB cells.

To determine the potential sequence over time of *Myc* amplification versus p53 loss-of-function, we performed qRT-PCR analysis at different time points and found that *Trp53* expression was downregulated after DIC 24 in *Ctdnep1*-cKO NPCs (Supplementary Fig. 12a), while at the later stage, an increase in *Myc* expression, was detected in *Ctdnep1-*cKO NPCs (Supplementary Fig. 12b, c). These data suggest that *Trp53* downregulation might occur prior to *Myc* gene overexpression or amplification during the transformation of *Ctdnep1*-cKO NPCs, alongside with the activation of MYC signaling caused by *Ctdnep1* loss. Thus, CTDNEP1 loss-of-function or deficiency leads to chromosome instability and aneuploidy, leading to p53 loss and *Myc* amplification during MB tumorigenesis.

### CTDNEP1 post-translationally modulates the activities of key mitotic checkpoint regulators

**Fig. 6:**
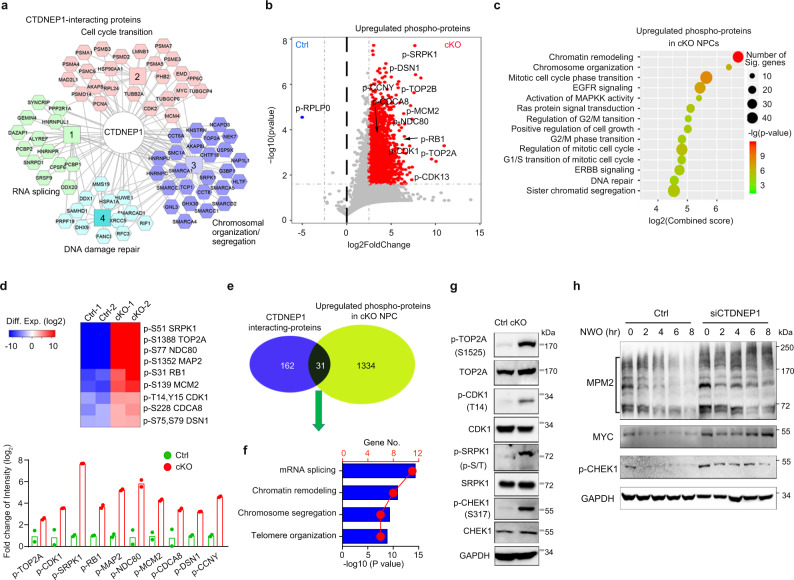
CTDNEP1 post-translationally modulates the activities of key regulators for chromosome decatenation and mitotic checkpoints. **a** CTDNEP1-interating proteins identified by mass spectrographic analysis in 293 T cells which was expressing HA-tag CTDNEP1. **b** Mass spectroscopy analysis of phosphorylated proteins in *Ctdnep1*-cKO NPCs (cKO) at DIC 10 compared with Ctrl NPCs. Two-tailed unpaired Student’s *t* test. **c** Pathway analysis of the most differentially upregulated phospho-proteins in *Ctdnep1*-cKO NPCs compared with wild-type NPCs. Fisher exact test. **d** Upper, representative phosphorylated proteins involved in cell-cycle progression that are enriched in *Ctdnep1*-cKO NPCs compared to control NPCs. Lower panel; the phosphorylation intensity of mitosis and chromosome segregation proteins in the NPCs detected by mass spectrometry. Data represent means, *n* = 2 independent experiments. **e** Venn diagram of CTDNEP1 binding proteins and phospho-proteins enriched in *Ctdnep1*-cKO NPCs compared to wild-type NPCs. **f** GO analysis of candidate CTDNEP1 interacting phospho-proteins in *Ctdnep1*-cKO NPCs. Fisher exact test. **g** Representative immunoblots from 3 independent experiments for p-TOP2A, p-CDK1, p-SRPK1 and p-CHEK1 in *Ctdnep1-*cKO and wild-type NPCs at late-stages. **h** Representative immunoblots from 3 independent experiments for the indicated phospho-proteins in D425 cells transfected with control siRNA or siCTDNEP1 after treatment with nocodazole for 14 h and sampled at indicated time points in fresh medium. NOW; nocodazole washout. Source data are provided as a Source Data file.

To better understand how CTDNEP1 loss may promote tumorigenesis, we sought to use proteomes to identify CTDNEP1 downstream interacting effectors through protein-protein interactions aside from the MYC protein. For this purpose, we performed immunoprecipitation with an antibody to CTDNEP1 and analyzed co-precipitated proteins from HEK293 cells by mass spectrometry. We identified 195 CTDNEP1 binding proteins, which regulate cell-cycle transition, RNA splicing, chromosome segregation/organization, and DNA repair processes (Fig. 6a). Co-immunoprecipitation assays validated the interaction between CTDNEP1 and a set of candidate binding partners such as TOP2A and SPRK1 in addition to MYC in a complex in D425 G3-MB cells (Fig. 3l and Supplementary Fig. 13a).

While little is known of CTDNEP1 substrate specificity as a protein phosphatase, we further sought to identify CTDNEP1 effects on phosphorylation, either direct or indirect, which could be related to tumorigenesis. Towards this end, we performed label-free mass spectrometry^51^ of wild-type and *Ctdnep1-*cKO NPCs at early-passage *Ctdnep1*-cKO NPCs to identify the upregulated phosphorylated proteins. When compared with control NPCs, nearly 3236 phosphorylated peptides, corresponding to 1365 proteins (fold changes >5; *p* < 0.05), were detected at significantly higher levels in *Ctdnep1-*cKO NPCs (Fig. 6b). Phospho-MYC peptides were not detected in the early-passage *Ctdnep1*-cKO NPCs, which might be due to a lower level of p-MYC prior to the MYC amplification seen at later stages (Fig. 5g). Gene ontology (GO) analysis indicated that the upregulated phospho-proteins are associated with cell-cycle progression and chromosome segregation (Fig. 6c). These proteins include critical regulators of chromosome decatenation and mitotic checkpoints for proper chromosome segregation such as DNA topoisomerase TOP2A and NCD80^52,53^; MCM2, which promotes DNA replication^54^; cell-cycle regulators SRPK1 and CDK1-3, which are serine-threonine protein kinases that regulate the G2-M transition and mitotic progression^55^; and the cell-cycle inhibitor RB1, which regulates transcription and the G1-S transition and is repressed by phosphorylation^56^ (Fig. 6d and Supplementary Fig. 13b–d).

We identified approximately 31 proteins as candidate direct targets or substrates of CTDNEP1. They were the CTDNEP1-binding proteins, and their phosphorylation were upregulated in *Ctdnep1*-cKO NPCs as compared to control NPCs (Fig. 6e). GO analysis showed the enrichment of processes critical for chromosome segregation and chromatin remodeling among the candidate proteins regulated by CTDNEP1 (Fig. 6f). These proteins interacted with CTDNEP1 and their phosphorylation was upregulated in CTDNEP1-deficient cells, suggesting that they are candidate direct substrates of CTDNEP1. Western blot analysis confirmed the increase in phosphorylation of a set of candidate CTDNEP1 substrates in *Ctdnep1*-cKO NPCs including TOP2A, CDK1, and SRPK1 (Fig. 6g), which regulate DNA replication, mitosis, RNA splicing, and chromosome separation^53,57,58^.

To investigate the effect of CTDNEP1 depletion on the phosphorylation of mitosis-associated proteins in MB cells, we examined the MPM2 epitopes that mark the phosphorylation of multiple M-phase-mitosis-promoting regulators during the mitotic metaphase^39,59^. Western blot analysis of control and *CTDNEP1*-knockdown in D425 G3-MB cells and SHH-MB DAOY cells indicated that CTDNEP1 depletion increased levels of mitotic phospho-proteins marked by MPM2 (Fig. 6h and Supplementary Fig. 14a), along with MYC upregulation. In addition, consistent with activation of DNA damage responses induced by *Ctdnep1* loss, we detected an increase in phosphorylation of a critical mitotic checkpoint kinase CHEK1 (p-CHEK1), which responds to DNA damage and regulates cell-cycle checkpoint signaling for cell survival^60,61^, in CTDNEP1-depleted MB cells and *Ctdnep1*-cKO NPCs (Fig. 6g, h). These results suggest that CTDNEP1 loss activates mitosis-associated checkpoint regulators to maintain mitotic homeostasis and cell survival.

To further determine the potential role of the CTDNEP1 effectors in genomic stability, we examined the activities of candidate CTDNEP1 substrates such as mitotic regulators CDK1, a key cell-cycle regulator^62^, and SRPK1, a serine/arginine protein kinase important for mitosis, chromatin reorganization, and tumor growth^63^, by constructing the vectors expressing phospho-mimetics, SRPK1-S51D and CDK1-T14E/Y15D (CDK1-ED), given that Y15 phosphorylation was also identified in our phospho-proteomic analysis. We found that overexpression of phosphomimetics, CDK1-ED or SRPK1-S51D, increased chromosomal abnormalities such as chromosome lagging and bridging at anaphase (Supplementary Fig. 14b, c). This supports a functional importance for CTDNEP1-regulated phosphorylation states of these putative substrates in maintaining proper chromosome segregation and genomic stability. Thus, our proteomics and phospho-proteomics suggest that CTDNEP1 modulates key regulators of chromosome decatenation and mitotic checkpoints for maintaining proper chromosome segregation and mitotic homeostasis.

### Co-targeting MYC and CHEK1 effectively inhibits the growth of MYC-driven MB tumors

**Fig. 7:**
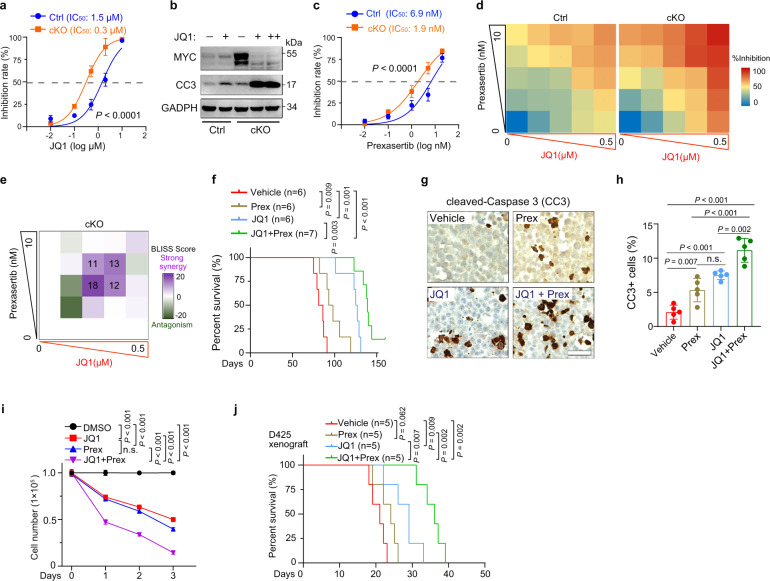
Combined targeting MYC and CHEK1 activities inhibits CTDNEP1*-*deficient tumor progression. **a** Cell viability of control and *Ctdnep1*-cKO NPCs treated with the indicated concentrations of JQ1 relative to vehicle-treated cells. Data represent means ± SD, n = 5 independent experiments. **b** Representative immunoblots from 3 independent experiments for MYC and cleaved Caspase 3 in control or *Ctdnep1*-cKO NPCs treated with JQ1 (0.5 μM: +; 1 μM: ++) or DMSO (−). **c** Cell viability of *Ctdnep1-*cKO NPCs and wild-type NPCs treated with prexasertib (Prex) for 3 days at indicated concentrations relative to vehicle-treated cells. **d**, **e** Heatmap showing the percentage of growth inhibition of *Ctdnep1-*cKO tumors and wild-type NPCs by combined treatment with JQ1 and prexasertib relative to vehicle-treated cells (**d**); Bliss score for JQ1 and prexasertib double titrations (**e**). *n* = 5 independent experiments. **f** Kaplan-Meier survival for *Ctdnep1-*cKO tumor-bearing allografts treated with vehicle, JQ1, prexasertib, or JQ1 and prexasertib combined treatment once/day for 2-weeks after transplantation at day 45. Log rank test. **g** Representative images of immunostaining for cleaved-Caspase 3 (CC3) in vehicle, JQ1, prexasertib, or JQ1 and prexasertib combined treated mice with *Ctdnep1-*cKO tumors 62 days after implantation. Red arrows CC3+ cells in the tumor section. **h** quantification of CC3+ cells in the tumor tissues of the indicated treatment groups. Scale bars: 50 μm. Data are means ± SD. *n* = 3 allografts; two-tailed Student’s *t* test. **i** Viability of D425 cells treated with JQ1 (1 μM), prexasertib (10 nM), or the combination relative to vehicle-treated cells. **j** Kaplan-Meier survival for MYC-driven D425 orthotopic xenografts treated with vehicle, JQ1, prexasertib or JQ1, and prexasertib once/day for 2-weeks after transplantation at day 10. Log-rank test. *n* = 5 independent experiments for **f**, **h**, and **j**. *n* = 3 independent experiments in **c**, **d**, **f**–**h**, and **j**. The data are presented as mean values ± SD; Two-way ANOVA for **a**, **c**, and **i**; two-tailed Student’s *t* test for **f**, **h**, **j**. Source data are provided as a Source Data file.

Since MYC and CHEK1 activities are upregulated in response to mutation or loss of *CTDNEP1* to promote cell growth and survival, we hypothesize that co-targeting of MYC and CHEK1 may inhibit the growth of CTDNEP1-deficient G3 tumors. We first utilized the BET inhibitor JQ1 and CDK7 inhibitor THZ1, both of which were shown to inhibit MYC expression^64,65^ and reduce cell proliferation in MB cells^66^. Indeed, treatment with JQ1 or THZ1 resulted in inhibition of *Ctdnep1*-cKO tumor cell proliferation (Fig. 7a and Supplementary Fig. 15a). JQ1 treatment also decreased MYC expression and elevated apoptosis marked by cleaved-caspase 3 (Fig. 7b). In view of upregulation of DNA damage repair and activation of p-CHEK1 for cell survival in *Ctdnep1*-deficient cells, we then treated *Ctdnep1*-cKO tumor cells with prexasertib, a selective inhibitor of the checkpoint kinase CHEK1 currently under multiple clinical trials; prexasertib disrupts DNA replication and prevents DNA damage repair, causing cell death by replication catastrophe^67,68^. This treatment similarly inhibited cell growth of *Ctdnep1*-deficient tumor cells (Fig. 7c). Importantly, combined JQ1 and prexasertib treatment of *Ctdnep1*-cKO tumor cells at reduced doses was more effective than either single agent (Fig. 7d). To determine if the effects of the two drugs on cell growth are synergistic, we applied the Bliss model to calculate synergy scores^69^ and classified the Bliss score >10 as synergistic^69^. The combined treatment with JQ1 and prexasertib elicited synergistic effects on the net cell growth in *Ctdnep1*-cKO tumor cells across a range of concentrations (Fig. 7e). To further determine the selectivity of the treatment on the growth of wildtype and *CTDNEP1-*deficient tumor cells, we treated control or *CTDNEP1-*knockdown D283 or MB-004 cells with JQ1 and prexasertib. *CTDNEP1* knockdown substantially increased the sensitivity of cells to drug combination treatment compared with control cells (Supplementary Fig. 15b–d), suggesting that this combined treatment more selectively inhibits the growth of *CTDNEP1* deficient tumor cells.

To assess the potential of MYC, CHEK1, or both for treating *Ctdnep1-*deficient tumors in vivo, we treated NSG mice orthotopically transplanted with *Ctdnep1*-cKO tumor cells daily with a single dose of JQ1 (50 mg/kg) or in combination with prexasertib (2 mg/kg), both of which are blood-brain barrier penetrant^70–72^, from day 45 to 60 post-transplantation. The mice bearing *Ctdnep1*-cKO NPC tumors treated with both JQ1 and prexasertib had an increased lifespan compared to the mice treated only with JQ1, prexasertib, or vehicle (Fig. 7f). The combined treatment also resulted in an increase in tumor cell death compared to vehicle or JQ1 treatment only (Fig. 7g, h), suggesting that the combined JQ1 and prexasertib treatment induces cell apoptosis to inhibit tumor growth.

To examine the effect of inhibition of MYC and CHEK1 on the growth of human G3 MB tumor cells, we treated MYC-driven D425 MB cells with JQ1 or prexasertib individually or in combination. Similarly, combined treatment synergistically enhanced inhibition of D425 cell growth in vitro (Fig. 7i and Supplementary Fig. 15e, f), and prolonged animal survival in orthotopic xenografts with D425 cells compared to vehicle or single drug treatment alone (Fig. 7j). In addition, combined MYC and CHEK1 inhibition substantially inhibited the cell growth in other MYC-amplified MB-004 and murine Myc-driven G3-like MB cells (Supplementary Fig. 15g), while the non-MYC-amplified cell lines such as D283 and DAOY were less sensitive to the combined treatment (Supplementary Fig. 15h). In contrast, treatment with inhibitors (galunisertib or LY‐364947) of TGF‐β receptor signaling^73^, which was elevated CTDNEP1-deficient NPCs (Fig. 5a), did not significantly alter the growth of *Ctdnep1*-cKO NPCs (Supplementary Fig. 15i, j). Together, these data suggest that combined inhibition of MYC and CHEK1 activities had a selective antitumor effect in G3-MB cells with CTDNEP1 deficiency or MYC amplification.

## Discussion

Although recent genome-wide studies have provided insight into somatically altered genes in MBs, identification and functional validation of cancer subtype specific-driving mutations remains enigmatic. *MYC* amplifications are a hallmark of highly aggressive G3 MB, yet the somatic mutations that trigger this phenomenon have remained poorly understood. By integrating the transcriptomic and genomic profiles from our newly diagnosed and publicly available MB cohorts^3,4,22,24^, we found that *CTDNEP1* mutations, which present predominantly in MYC-driven MBs, define a specific subset of aggressive MB tumors. In contrast to many mouse models of MYC-driven G3 MB which require MYC overexpression and additional loss of p53 function^12,13^, here we demonstrate that ablation of a single gene, *Ctdnep1*, is sufficient to trigger MYC signaling activation and MYC overexpression or amplifications, while promoting malignant transformation of cerebellar NPCs into MYC-driven MB tumors. CTDNEP1-deficiency-induced tumors resemble the histopathological, transcriptomic, and clinical features of human G3 MB counterparts, suggesting that CTDNEP1 is a potent tumor suppressor in the highly aggressive MYC-driven G3 MBs.

MYC has been shown to tether to the nuclear pore specifically in cancer cells^74^, and the enrichment of activating pS62-MYC at the nuclear periphery promotes its stability and oncogenic activity^15–17^. Our proximity ligation assays indicate that CTDNEP1 is able to interact with MYC and p-MYC at the nuclear periphery, suggesting that CTDNEP1 might regulate MYC activity directly, although CTDNEP1 may also regulate MYC oncogenic activity through other indirect pathways. Exactly how CTDNEP1 binding regulates MYC oncogenic activity remains to be determined. Nonetheless, we find that clinically-identified mutations in CTDNEP1 disrupt its phosphatase activity, resulting in an increase of pS62-MYC and MYC stabilization. Thus, our data suggest that the nuclear-envelope-enriched CTDNEP1 phosphatase activity might at least in part curtail the MYC protein level and its oncogenic activity.

MYC-driven G3 MBs include tumors with MYC pathway activation and MYC overexpression or amplification, exhibiting a core attribute of MYC signaling activation. In addition to the observation that half of CTDNEP1-deficient patients have *MYC* amplifications, we found that CTDNEP1 deficiency results in an increase in MYC stability and activation of MYC signaling. These data indicate that CTDNEP1 mutations or deficiency may augment MYC oncogenic activity through MYC signaling activation and MYC amplification, suggesting that CTDNEP1 loss-of-function mutations might be generally relevant to MYC-driven G3 MB. Notably, since CTDNEP1 mutations or deletions have also been identified in other cancers (Supplementary Fig. 16), CTDNEP1 might play a broader role in cancer formation serving as a molecular link regulating MYC activity and expression across different cancer types.

**Fig. 8:**
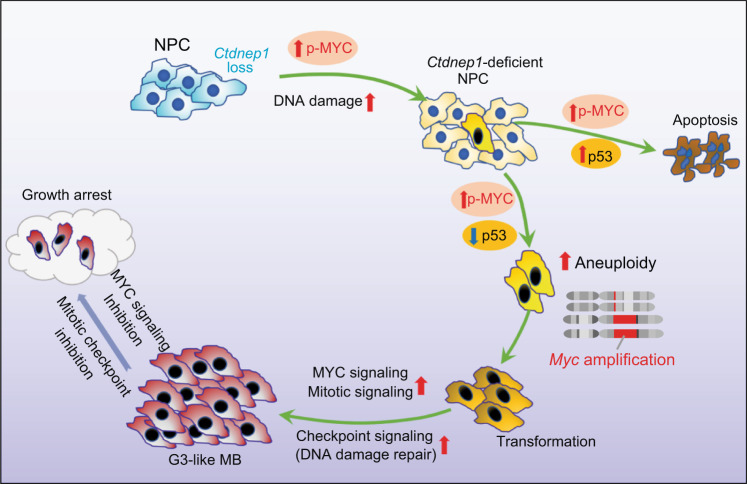
A schematic model for G3 MB transformation induced by CTDNEP1-deficiency. CTDNEP1 depletion or mutation in neural stem/progenitor cells (NPCs) results in MYC activation along with DNA damage and increased p53 levels, leading to apoptosis at the early stages. However, a population of CTDNEP1-deficient NPCs acquires the selective fitness advantage to survive by inducing p53 loss or downregulation and triggers genomic instability and aneuploidy with *Myc* gene amplifications. Together with p53 loss and *Myc* amplification, the increased CHEK1-mediated DNA damage repair and mitotic checkpoint signaling further contributes to the transformation of CTDNEP1-deficient NPCs into malignant G3-like MBs. Targeting MYC and mitotic checkpoint signaling with JQ1 and prexasertib, respectively, inhibits the growth of the CTDNEP1-deficient G3-like MBs.

*Ctdnep1* ablation in Nestin+ NPCs causes extensive cell death in the developing brain, which may contribute to animal death prior to tumor formation, suggesting that additional genetic, epigenetic, and other molecular events are necessary prior to full transformation. We observed an increase in DNA damage responses and p53 upregulation at the early stage in response to *Ctdnep1* ablation in NPCs, suggesting that acute *Ctdnep1* deletion may induce DNA damage and p53 upregulation-mediated cell apoptosis. However, a population of *Ctdnep1-*ablated cerebellar NPCs at later stages exhibit p53 downregulation, acquire chromosomal aneuploidy and *Myc* amplification, and are eventually transformed into MYC-driven MB tumors. Downregulation of p53 appears to occur during selection of clones with MYC activation or upregulation, which represses p53 expression^43^. Consistent with this, we show that p53 loss-of-function by overexpressing dominant-negative p53 can accelerate the tumorigenesis of freshly isolated *Ctdnep1*-deficient NPCs in allografts. Yet, it remains to be defined whether CTDNEP1 can directly target p53. Nonetheless, we found that sustained CTDNEP1-depletion can induce genome instability and aneuploidy at a late passage of *Ctdnep*1-cKO NPCs, resulting in p53 downregulation and *Myc* amplification, which have been shown to promote oncogenic transformation^75,76^. These observations suggest that CTDNEP1-loss-induced MYC activation and the subsequent p53 deficiency might contribute to MYC amplification, and that CTDNEP1 might regulate the MYC pathway through both direct and indirect mechanisms (Fig. 8).

Intriguingly, all tumors from patients with *CTDNEP1* mutations appeared to exhibit loss-of-heterozygosity (LOH) for the other allele of *CTDNEP1*. This suggests that *CTDNEP1* may exhibit bi-allelic inactivation in patient tumors, which is typical of many tumor suppressors^77,78^. Since *CTDNEP1* and *TP53* are tightly linked on chromosome 17p, one copy of *TP53* might be lost along with an allele of *CTDNEP1*. *CTDNEP1* loss-of-function due to mutation in the other allele could drive increased proliferation and genome instability due to MYC upregulation. Importantly, independent LOH for *TP53*, subsequent to CTDNEP1 loss-of-function, could further promote transformation and tumorigenesis by resulting in deficient cell-cycle arrest and apoptosis in cells with increased DNA damage and genomic instability^79,80^.

Our studies provide experimental evidence demonstrating that clinically relevant mutations in CTDNEP1 promote MYC-driven G3-MB tumorigenesis by inducing MYC activation, genomic instability, and mitotic infidelity. While the exact mechanisms underlying chromosome instability and MYC amplification caused by mutations in *CTDNEP1* remain to be determined, increased MYC oncogenic activity in CTDNEP1-deficent cells may trigger genomic instability, which could potentially lead to MYC gene amplification^9–11^. Our data indicate that a potential key target of CTDNEP1 is MYC, which can be regulated by CTDNEP1-catalyzed dephosphorylation of S62, resulting in MYC signaling activation. In addition, CTDNEP1 may also regulate genomic stability in an MYC-independent manner by maintaining proper DNA replication and mitotic exit. Our unbiased proteomic analyses identify a set of potential CTDNEP1-interacting effectors, including mitotic regulators critical for DNA replication, chromosome decatenation, and mitotic checkpoints such as TOP2A, MCM2, CDK1, SRPK1, and RB1^53,56,57^. Importantly, we found that overexpression of phospho-mimetics of candidate CTDNEP1 targets such as CDK1-ED or SRPK1-S51D induced genomic instability and mitotic errors, consistent with a role of CTDNEP1 for mitotic fidelity in other contexts^81^. Given that transient genomic instability can drive tumorigenesis^82^, our data support the possibility that CTDNEP1 regulates the activity of mitotic regulators, including MYC, to maintain genomic stability at least in part through the effects on their post-translational modifications. Thus, the tumor suppressor activity of CTDNEP1 may be exerted in part by inhibiting MYC activity while maintaining cell-cycle homeostasis and genomic stability.

Given increased chromatin accessibility at the *Myc* locus only in late-stage *Ctdnep1*-ablated NPCs, our data indicate these two events, MYC stabilization and *Myc* amplifications due to the gain of *Myc* amplicons induced by CTDNEP1 loss, occur in a sequence that may eventually drive the increased MYC overexpression and NPC transformation through a positive feedback loop. Our data indicate that the growth of *Ctdnep1*-deficient tumor cells depends on MYC levels, which is in keeping with a critical role of MYC in tumor cell proliferation and chromosome instability^40,44–46^. Thus, the loss of CTDNEP1 not only leads to increased MYC levels by stabilizing it, but also provides a selective advantage to cells that can express even greater levels of MYC due to increased copy numbers and chromatin accessibility (Fig. 8).

Our data showing upregulation of MYC and CHEK1 may present a potential therapeutic vulnerability. Importantly, LOH of *CTDNEP1* in the tumor may increase the therapeutic window for combined treatment with MYC and CHEK1 inhibition. Such a dual inhibitory strategy could be especially beneficial in the treatment of MYC-driven MB, given that elevated MYC levels have been shown to sensitize cancer cells to the inhibition of mitosis and checkpoint signaling by increasing apoptosis^65,83^, since CHEK1 signaling activation is correlated with cell survival and poor prognosis in MYC-driven MB^84^. We find that JQ1 suppresses *MYC* transcription and can synergize with CHEK1 inhibitor prexasertib to suppress tumor growth more effectively than either single agent alone, while prolonging survival in the animals bearing CTDNEP1-deficient MYC-amplified MB tumors. Notably, prexasertib is currently being evaluated in clinical trials, including the treatment of pediatric malignancies^54,67,68^. Patients with MB tumors such as highly aggressive MYC-driven MB commonly have DNA replication stress and pronounced DNA damage responses, resulting in upregulation of checkpoint signaling^85^. Prexasertib treatment may induce cell apoptosis by inhibiting the repair of DNA damage caused by *CTDNEP1* deficiency. Thus, combined targeting of MYC and checkpoint regulators such as CHEK1 might serve as a therapeutic means to improve outcomes in treatment of aggressive G3 MBs with *CTDNEP1*-deficiency. Further, these vulnerabilities underscore a critical role of CTDNEP1 in suppressing malignant transformation of the highly aggressive G3 MBs by inhibiting MYC oncogenic activity while maintaining mitotic fidelity and genomic stability.

## Methods

### Animals

Mice carrying *Ctdnep1* floxed alleles (Acc. No. CDB0564K) were generated as described^20^. *Ctdnep1*^f/f^ mice were crossed with mouse lines carrying Cre recombinase driven by the nestin promoter (Nestin-Cre^+/−^) to generate *Ctdnep1*-cKO (*Ctdnep1*^f/f^; Nestin-Cre^+/−^) and controls (*Ctdnep1*^f/+^; Nestin-Cre^+/−^ or *Ctdnep1*^f/f^). In this study, mice of either sex (male and female) were analyzed, and littermates were used as controls unless otherwise indicated. Mouse strains were generated and maintained on a mixed C57BL/6; CD-1 background and fed (4 or less mice per cage) in a vivarium. Immunodeficient NOD scid gamma (NSG) mice were provided by Cincinnati Children’s Hospital Medical Center (CCHMC) animal core. Mice were housed at room temperature (20–23 °C) with a 12-h light–dark cycle set with lights on from 06:00 to 18:00 and with humidity between 30–80%. The animal studies were approved by the IACUC (Institutional Animal Care and Use Committees) of the Cincinnati Children’s Hospital Medical Center, USA. For euthanasia of neonates and adult mice, we performed CO_2_ overdose followed by cervical dislocation. Animal survival endpoint is the date of the animal that died or was euthanized according to animal use guidelines. Animals were removed from the study and were harvested when they exhibited >20% decrease in body weight based on the IACUC protocol. The limits of endpoints were not exceeded in any of the experiments. All studies complied with the animal use guidelines and ethical regulations.

### MB tumor data, *CTNDEP1* mutation, and survival analysis

The use of tumor samples was approved by individual institutional review boards (IRB) from Cincinnati Children’s Hospital and Children’s Hospital of Fudan University. All data of newly diagnosed medulloblastoma tumors of the Asian cohort and subgroup analysis (89 tumor samples) were described in the previous study^22^. To identify independent *CTNDEP1* mutations in publicly available datasets from reported cohorts^4,5,7,14,23^, CBTTC cohort (https://cbttc.org/), and the Asian cohort, we performed quality control (QC) based on the unique patient IDs and clinical information including gender (male and female), age, tumor subgroup, and histological subtype, as well as copy number variation (CNV) features, to ensure the removal of sample duplicates among different cohorts and identify individual patients with *CTNDEP1* mutations.

For the survival analysis based on CTDNEP1 expression, in view of the unequal expression *CTDNEP1* in each subgroup, we plotted the density curves of MB cohorts (total 612 MBs) from publicly available datasets for CTDNEP1 expression and survival analysis and found that the patient density across different MB subgroups is enriched between *CTDNEP1* expression levels 7.67 and 7.98 (log2 intensity) (Supplementary Fig. 3d). We, therefore, set up the threshold that 35% highest expression samples were stratified into the high group (log2 intensity >7.98) and the 35% lowest expression samples as the low group (log2 intensity <7.67) for all MB samples (total 612 MBs) from publicly available datasets^3^ for CTDNEP1 expression and survival analysis. Namely, the highest 35% and lowest 35% patient populations are established as the cutoff to compare patient survival within two subgroups for all patients or each subgroup. Overall survival curves were plotted with the Kaplan-Meier method and compared by using a two-sided log-rank test.

### DNA methylation array processing and CNV analysis

MB sample DNA methylation profiling was performed using the Infinium CytoSNP-850K v1.2 BeadChip array (EPIC 850 K array) according to the manufacturer’s instructions (Illumina). The subtypes of human MB samples were defined based on published annotations^5,7^. All DNA methylation analyses were processed in R v.3.3.0 (R Development Core Team, 2016) and the detailed information was described previously^4^. MB clusters were defined as WNT, SHH, G3, and G4 based on classification using previously described 48 CpG signatures^5,7^. CNV analysis of the MB from 850 K methylation array data was performed using the conumee Bioconductor package v.1.4.0. A set of 10 control samples methylation array data displaying a balanced copy-number profile was used for normalization.

### Primary NPC isolation and culture

Primary cerebellar NPCs were isolated according to the previous report^86^ with minor modification from *Ctdnep1*-cKO (*Ctdnep1*^f/f^;Nestin-Cre^+/−^) and control mice, which carry a *Rosa26*:ccGFP reporter. Briefly, isolated cells were cultured in complete NPC medium (Neurobasal medium containing B27 supplement, N2 supplement, 2 mM L-glutamine, 20 ng/ml EGF, 20 ng/ml bFGF, 2 mg/ml Heparin and 50 μg/ml BSA). NPC spheres were dissociated at a diameter of 100–200 μm and were changed with complete NPC medium every 3 days to maintain cell metabolic activity. We defined three stages of NPCs in vitro culture, including: early stage (1–20 days in culture), mid-stage (21–50 days in culture), and late-stage (>51 days in culture). In addition, freshly isolated NPCs from *Ctdnep1*-cKO animals were also transduced with retroviruses expressing dominant-negative p53 and transplanted into the cerebellum of NSG mice.

### Cell line culture

Medulloblastoma cell lines D425, D283, D458, and DAOY were obtained from American Tissue Culture Collection (ATCC, Rockville, MD, USA). MB-004 and murine G3 MB lines were provided by Dr. Martine Roussel. Cells were cultured in DMEM/F12 media with 10% FBS, 2 mM l-glutamine, and 1% Penicillin/Streptomycin. HeLa, U2OS, and HEK293T cells lines from ATCC were maintained in DMEM with 10% fetal bovine serum, 2 mM l-glutamine, and 1% penicillin/l streptomycin at 37°C in an atmosphere of 5% CO_2_. To investigate chromosomal stability, we used U2OS cells as they are a widely used cell model for verifying the functional effect of targets on chromosome segregation as described in the previous studies^87,88^.

### Vectors, RNA interference, QPCR, and lentivirus production

Wildtype CTDNEP1 and mutant D67N, D69N, and L72H were cloned with Myc-tag into a pLVX-puro vector (Addgene). c-Myc and mutant S62E, D62A were amplified and then cloned into pcDNA3.1-3xFLAG-CMV. shRNAs against CTDNEP1 were designed at https://rnaidesigner.thermofisher.com/rnaiexpress/ (Supplementary Data 1) and then cloned into pGreen-puro vector. siRNA targeting CTDNEP1 or control siRNA was ordered from Sigma-Aldrich (www.sigmaaldrich.com, siRNA ID: SASI_Hs01_00188422 and SASI_Hs01_00188427). siRNA interfering CTDNEP1 was performed using Lipofectamine RNAiMAX Transfection Reagent (Qiagen) according to the manufacturer’s instructions. For the QPCR, 1 μg RNA was used to generate the 1st stand of the cDNA using iScript Reverse Transcription Supermix (BioRad, #1708 841). QPCR was performed using SYBR green PCR mix (BioRad) and the primers as listed (Supplementary Data 1).

To produce lentiviruses, HEK293T cells were co-transfected with shRNA or GFP vector packaging using Lipofectamine 3000 reagent (Life Sciences). Supernatants were collected and filtered at 48 and 72 hr following transfection. Viral supernatant was concentrated by centrifugation at 25,000 rpm for 2 hr at 4°C and used to infect cells (MOI = 5) overnight in the presence of 10 μg/mL polybrene. Cells were selected and maintained with puromycin (2 μg/ml). Gene expression was verified by western blot or real-time PCR.

### Cell proliferation, colony formation, and neurosphere formation assays

Cell proliferation was measured by CCK-8/WST-1 or EdU assays. For EdU assays, we used the Click-iT™ Plus EdU Cell Proliferation Kit for Imaging (Invitrogen). After EdU incubation, cells were fixed with 4% paraformaldehyde and permeabilized with 0.3% Triton X-100, and EdU detection was performed according to the manufacturer’s instructions. Nuclei were counterstained with Hoechst 33342 reagent. At least 500 nuclei were counted in triplicate, and the number of BrdU-positive nuclei was recorded. For colony-formation assays, Ctrl and shRNA-infected cells were seeded in six-multiwell plates. After 2 weeks, cells were fixed with 4% PFA and stained with crystal violet. For neurospheres, 1000 cells/ml were seeded in low attachment 96-multiwell plates in DMEM with neurosphere medium as previously described^13,89^. The number of neurospheres was counted and captured images after 10–15 days.

### Tissue processing, antibodies, immunostaining, and immunoblotting

Mouse brains were dissected and fixed overnight in 4% (w/v) paraformaldehyde and processed for cryosectioning or paraffin embedding and sectioning described previously^8^. Briefly, for the immunostaining, cryosections or pre-deparaffinized tissue sections were firstly blocked 1 h by block solution [PBS with 5% v/v normal goat serum (Sigma-Aldrich) and 0.3% v/v Triton X-100) and incubated overnight in primary antibodies diluted in antibody dilution solution [PBS with 5% v/v normal goat serum (Sigma-Aldrich)]. After washing with PBS 5 times, sections were then either incubated overnight with the biotinylated goat anti-mouse IgG antibody (Vector Laboratories, BA-9200), followed by using the ABC avidin/biotin method to visualize staining signals under light microscopy with the peroxidase/diaminobenzidine (DAB) method, or incubated with corresponding fluorophore-conjugated secondary antibodies (donkey anti-rabbit IgG Alexa Fluor 488, Jackson ImmunoResearch, Cat#711-545-152, 1:500; donkey anti-mouse IgG Alexa Fluor 488, Jackson ImmunoResearch, Cat#711-545-150, 1:500; donkey anti-rabbit IgG Alexa Fluor 594, Jackson ImmunoResearch, Cat#711-585-152, 1:500; donkey anti-mouse IgG Alexa Fluor 494, Jackson ImmunoResearch, Cat#711-585-150, 1:500), and DAPI (Millipore Sigma; Cat#D9542, 300 nM) under fluorescent microscopy.

For cell immunostaining, spheres were fixed with 4% PFA for 10 min and washed five times with PBS and dehydrated with 30% sucrose overnight, then blocked with OCT frozen embedding media (CRYO-4; Polarstat Inc.) and cryosectioned at 12 μM thickness. For adherent cells, cells were planted on the coverslips and fixed with 4% PFA for 10 min and washed five times with PBS. Then placed the sections or coverslips with cells in blocking solution for 30 min. We incubated primary antibodies in blocking solution with proper dilutions and stained cells for 1 h at room temperature. For BrdU staining, cells or tissue sections were denatured with 0.1 N HCl for 1 h in 37 °C water bath. After denaturation, sections were neutralized with 0.1 M Borax, pH 8.5 (Sigma) for 10 min. Sections were washed with 1× PBS three times and blocked with 5% normal donkey serum (Sigma-Aldrich) in wash buffer for 1 h at room temperature. Mouse-anti BrdU (BD Bioscience, 1:500) antibody was used to label BrdU overnight at 4 °C. DAPI counterstain was included in the final washes before the samples were mounted in Fluoromount G (SouthernBiotech) for microscopy. Tissue or cell images were quantified in a blinded manner. All immunofluorescence-labeled images were captured using a Nikon C2 + confocal microscope.

Primary antibodies used were: Nestin (Mouse, Abcam; Cat#ab22035, 1:500), Ki67 (Rabbit, Thermo Fisher; Cat#MA5-14520, 1:1000), BrdU (Mouse, BD Bioscience; Cat#347580, Abcam; Cat#ab6326, 1:500), Cleaved Caspase 3 (Rabbit, Cell Signaling; Cat#9661, 1:500), c-Myc (Rabbit, Cell Signaling; Cat#5605 S, 1:1000), γH2A.X (Rabbit, Cell Signaling; Cat# 9718 S, 1:1000), p53 (Rabbit, Cell Signaling; Cat# 2524 S, 1:1000), p-S15 p53 (Rabbit, Cell Signaling; Cat#9284, 1:1000), GAPDH (Mouse, Thermo Sci; Cat# 39–8600, 1:5000), phosphor-Ser/Thr-Pro MPM2 (Mouse, Millipore Sigma; Cat#05-368, 1:1000), p-S62 c-Myc (Rabbit, Abcam; Cat#ab51156, 1:1000), p-S1525 TOP2A (Rabbit, Cedarlanelabs; Cat#E-AB-21933, 1:1000), TOP2A (Rabbit, Proteintech; Cat#20233-1-AP, 1:1000), p-S317 Chk1 (Rabbit, Cell Signaling; Cat#12302, 1:1000), Chk1 (Rabbit, Proteintech; Cat#25887-1-AP, 1:1000), Cdc2 (Rabbit, Cell Signaling; Cat#9116 T, 1:1000), p-T14 Cdc2 (Rabbit, Cell Signaling; Cat#2543 S, 1:1000), SRPK1 (Rabbit, BD biosciences; Cat#611072, 1:1000), HA-Tag (Mouse, Cell Signaling; Cat#2367, 1:1000), DYKDDDDK-Tag (Mouse, Thermo Fisher, Cat# MA1-91878, 1:1000), Phosphor-Ser/Thr (Rabbit, Abcam; Cat#ab117253, 1:1000) and GFAP (Goat, Santa Cruz; Cat#sc-6170, 1:500). All immunofluorescence-labeled images were acquired on a Nikon C2 confocal microscope.

For Western blot, cells were lysed with RIPA lysis buffer (Millipore, 20–188) supplemented with cOmplete phosphatase and protease inhibitor cocktail (MilliporeSigma/Roche 11836153001). Protein concentration of each sample was determined by BCA assay using the BCA kit (Thermo Fisher, 23227) according to the manufacturer’s instructions, and equal amounts were loaded and separated by 12% SDS-PAGE gel. PVDF membrane (Millipore) was used for gel transfer and the membrane was probed with primary antibodies as indicated, followed by secondary antibodies conjugated with biotinylated goat anti-mouse IgG antibody (H + L) (Vector Laboratories, BA-9200). The signal was detected with SuperSignal West Pico/Femto Chemiluminescent Substrate (Thermo Scientific, 34577).

### Anaphase, mitotic exit, karyotype analysis, and FISH experiment

For anaphase analysis of the cultured NPCs, all cells were planted in the coverslips precoated by poly-l-lysine (Sigma-Aldrich, St. Louis, MO, P5899) at 100 μg/ml 30 mins and coated with laminin (Sigma-Aldrich, St. Louis, MO, L4544) at 50 μg/ml for 30 mins. For capturing the anaphase, NPCs were synchronized at the G2-M boundary by nocodazole (Sigma-Aldrich, St. Louis, MO, M1404) at 100 ng/ml for 4 h and released to a fresh medium and continued to culture 10–60 mins. Coverslips were fixed with 4% PFA for 15 mins at 5 min interval and stained with DAPI for DNA contents.

For mitotic exit assays, prometaphase-arrested cells were obtained by performing a double thymidine (2 mM; Sigma-Aldrich, St. Louis, MO) block (18 hr each, separated by a 6 hr incubation in fresh medium) followed by release into fresh medium containing nocodazole (100 μg/ml; Sigma-Aldrich, St. Louis, MO) and incubation for 12 or 14 h. Release from prometaphase arrest was obtained by washing detached cells twice with PBS and twice with fresh medium, followed by incubation in fresh medium. Cells in G1 were obtained after 120 min incubation from prometaphase release. The probes for mouse *Myc* and chromosome 15 (RP23-275E10) were purchased from Empire Genomics for Fluorescence In Situ Hybridization (FISH). FISH experiments and G-band karyotype analysis of mouse NPCs and tumors were analyzed by Cincinnati Children’s Hospital histology core (https://www.cincinnatichildrens.org/service/d/diagnostic-labs/cytogenetics).

### Western blotting

Tumor tissues were lysed in modified RIPA buffer (50 mM Na-Tris, pH 7.4, 150 mM NaCl, 1% (v/v) NP-40, 0.25% sodium deoxycholate, 1 mM dithiothreitol, 10 mM NaF, 1 mM active sodium vanadate, 1 mM PMSF and 1× a cocktail of cOmplete protease inhibitors (Roche Applied Science) and centrifuged at 17,000 × *g*. for 15 min at 4 °C. After the determination of protein concentration (Bio-Rad), the lysates were separated by 4–12% SDS-PAGE. Bands were visualized with secondary antibodies conjugated to horseradish peroxidase (Bio-Rad) and ECL western blotting detection reagents (Pierce) per the manufacturer’s instructions.

### Proteomic profiling

CTDNEP1 binding protein was analyzed by immunoprecipitation for CTDNEP1-myc in CTDNEP1 enforced expression HEK293T cells and followed by mass spectrometry at the Fudan University core facility. Phospho-proteome were processed according to protocols adapted from previous studies^90^ and performed using label-free quantitative proteomics technology (Clinproteomics Co., Ltd).

### Transplantation of *Ctdnep1*-deficient tumors and drug treatment in vivo

*Ctdnep1*-deficient Tumors cells were subcutaneously injected into eight-week-old female athymic BALB/c nude mice or NSG mice. Tumors will be harvested after 6–10 weeks and quantified. 1 × 10^5^ NPCs in a 5 μl with 2 μl matrigel were stereotactically injected into the NSG mouse cerebellum. Animals were monitored weekly and euthanized when they showed signs of brain tumor. The mouse brain tissue with tumor was embedded in paraffin and sectioned at a thickness of 5 μm for H/E and immunohistochemistry assays. *Ctdnep1* cKO tumors exhibit the large cell/anaplastic morphology observed in MYC-driven Group 3 MB, which was confirmed by a neuropathologist Dr. Christina Fuller. The NSG mice with *Ctdnep1*-cKO NPC tumors in the cerebellum were randomized into different groups and administered Prexasertib (15 mg/kg) and/or JQ1 (50 mg/kg) or vehicle (10% DMSO in 10% HP-β-Cyclodextrin, Sigma) on alternating days via intraperitoneal injection for 14 days^64^.

### Whole genome sequencing (WGS) and single nucleotide variant calling and copy number variations (CNV) analysis

WGS-derived raw image files were processed by DNBseq basecalling Software for basecalling with default parameters and the sequence data of each individual is generated as paired-end reads as FASTQ format. Single nucleotide variant analyses conducted using the Genome Analysis Tool Kit (GATK)(https://gatk.broadinstitute.org/hc/en-us). Briefly, the fastq data files from mouse WGS were mapped to mouse genome (mm10) by Burrows-Wheeler Aligner (BWA) in the GATK4 module. HaplotypeCaller was used to call the single nucleotide variants (SNV) and insertion deletions (Indel). All variants were removed the SNPs and then annotated by ANNOVAR. CNVs were called using SOAPcnv software. Based on the result of SOAP alignment, the depth of each base should be calculated and standardized by the mean depth of its chromosome to calculate the copy number variation. CNVs were detected by the following steps: (1) DNA sequences were separated into fragments according to the depth of each base from the alignment results; (2) The *P* value was calculated for each fragment to estimate its probability to be a CNV; (3) The fragments that passed the criteria (fragment length longer than 2 kb, *P* value < = 0.35, mean depth less than 0.5 or >2.0) were kept as CNVs. The mapped bam files from WGS were used for CNV analysis. We followed the somatic copy number variation pipeline from GATK4 CNV (https://github.com/ding-lab/gatk4wxscnv). The final segment ratio files with CNV type annotation for all NPC and tumor samples were further annotated by AnnotSV.

### Phosphatase and cell cycle assays

The phosphatase activity of CTDNEP1 was analyzed using *p-*NPP (*para*-nitrophenyl phosphate, Sigma) as described^19^. Briefly, 5 μg CTDNEP1 and its mutations plasmids were introduced into 1 × 10^6^ 293 T cells and the CTDNEP1 proteins were purified by immunoprecipitation using anti-Myc-tag-beads (Sigma and Cat# A7470). The reaction mixture (20 μl) contained 50 mM Tris-acetate (pH 5.5), 10 mM MgCl_2_, 0.5–50 mM *p-*NPP, and CTDNEP1 proteins incubated at 37 °C for 20 min. 80 μl of 0.25 N NaOH was added to stop the reaction. Release of *p*-NP (*para*-nitrophenol) was determined by measuring the absorbance at 410 nm.

For the dephosphorylation of c-Myc, we added the CTDNEP1 proteins purified by Myc-affinity beads to the 50 μl D425 cell lysate which contained Tris-acetate and MgCl_2_ incubated 1 h and the phosphorylation of c-Myc was determined by immunoblotting.

For cell-cycle analysis, the CycleTEST™ PLUS DNA Reagent Kit (BD 340242) used to stain cell nuclei according to the manufacturer’s instructions. Flow cytometry was conducted using BD FACSCanto Flow Cytometer. Raw data were analyzed using FlowJo software. The histogram of cell-cycle distribution was generated from at least 10,000 events per sample.

### RNA-seq and differential gene expression analysis

Total RNA was extracted from fresh cells or frozen tissue using TRIzol (ThermoFisher) and purified by RNeasy kit (www.qiagen.com). Quality of total RNA for each sample was checked on an Agilent Bioanalyzer 2100 RNA Nano chip (Agilent). RNA samples with RNA integrity numbers at least 7 were used for library preparation (polyA enrichment) and sequenced by Novogene (https://en.novogene.com/) or BGI (www.bgi.com) with 150 base pair paired-end reads.

To examine transcriptomic differences, cDNA reads were aligned to hg19 for human cells or mm10 for mouse cells using TopHat2 alignment to generate bam files^42^. Unnormalized gene read counts were generated using Cufflinks (http://cole-trapnell-lab.github.io/cufflinks/). Differentially expressed genes were normalized and analyzed using the Cuffdiff.

### Assay for transposase-accessible chromatin using sequencing (ATAC-Seq)

ATAC-seq assays were performed as previously described^38^. Briefly, we isolated nuclei of ~50,000 cells in a cold lysis buffer (10 mM Tris-HCl, pH 7.4, 10 mM NaCl, 3 mM MgCl2, 0.1% IGEPAL CA-630). After spinning down at 500 × *g* for 10 min at 4 C, nuclei were resuspended in transposition mix containing TD (2× reaction buffer), TDE1 (Nextera Tn5 Transposase) at 37 °C for 30 min. Immediately following transposition, DNA were purified using a Qiagen MinElute PCR Purification Kit. Transposed DNA fragments were subsequently amplified and the amplified library was purified using Qiagen MinElute PCR Purification Kit. Libraries were generated using the Ad1_noMX and barcoded primers and were amplified for 11 total cycles. Libraries were purified with AMPure beads (Agencourt) to remove contaminating primer dimers and were sequenced on the Illumina HiSeq 2500 with 75 bp single-end reads.

Reads of ATAC-seq data were aligned to rn5 genome using Bowtie with the following options:–best–chunkmbs 200 (http://bowtie-bio.sourceforge.net). Peak calling was performed using Model-based analysis of MACS version 2.12 (https://github.com/ taoliu/MACS) with specific parameters without the prebuilt model:–shift 75–extsize 150–nomodel–call-summits–nolambda– keep-dup all -p 0.01, to call peaks, which extend and shift the fragments to get the region cut by the Tn5 sites. We calculated the peak_RPKM, then GSEA (v2.2.0) was used to analyze the enrichment of signature gene sets.

### Statistical and survival analysis

All analyses in this research were performed using Microsoft Excel, GraphPad Prism 8 (San Diego California, https://www.graphpad.com) or RStudio (https://www.rstudio.com/ and R v.3.4.0). We use the “cor” function in R to calculate the Pearson correlation coefficient. Association between CNV and somatic mutational events were performed using Fisher Exact Test (R), FDR was used to adjust multiple tests. The Fisher’s exact test was used to determine the significance of gene mutations that are specifically enriched in G3-MB compared with other MB subgroups. Statistical significance was determined using two-tailed Student’s *t* tests as indicated. One-way ANOVA test was performed by multiple comparisons following Turkey’s ranking tests when comparing multiple groups. Data are shown as mean ± SD (error bars). Values of *p* < 0.05 denoted a statistically significant difference. Quantifications were performed from at least three experimental groups in a blinded fashion. The *n* value was defined as the number of experiments that were repeated independently with similar results. No statistical methods were used to predetermine sample sizes, but our sample sizes are similar to those generally employed in the field. No randomization was used to collect all the data, but data were quantified with blinding.

### Reporting summary

Further information on research design is available in the Nature Portfolio Reporting Summary linked to this article.

## Supplementary information

Supplementary Information

Description of Additional Supplementary Files

Supplementary Data 1

Reporting Summary

## Data Availability

All high-throughput data generated in the paper are deposited in the NCBI Gene Expression Omnibus (GEO). The accession number is GSE145921. The mass spectrometry proteomics datasets are deposited in ProteomeXchange (Identifier: PXD019067). Source data are provided with this paper.

## Source data

Source Data

## Code availability

No custom code was used in this study. Open-source algorithms were used as detailed in analysis methods, including Enrichr (https://maayanlab.cloud/Enrichr/), Galaxy (https://usegalaxy.org/), GSEA (https://www.gsea-msigdb.org/gsea/index.jsp), AltAnalyze (http://www.altanalyze.org/) and Toppgene (https://toppgene.cchmc.org/), Tophat (http://tophat.cbcb.umd.edu), Genome Analysis Tool Kit (GATK) (https://gatk.broadinstitute.org/hc/en-us), ToppGene Suite (https://toppgene.cchmc.org/), reference genome mm10 (https://hgdownload.soe.ucsc.edu/downloads.html).
