# Supplementary material for: Loss of phosphatase CTDNEP1 potentiates aggressive medulloblastoma by triggering MYC amplification and genomic instability
Source: Nat Commun. 2023 Feb 10;14:762. doi: 10.1038/s41467-023-36400-8 (PMC9918503; doi:10.1038/s41467-023-36400-8)
Supplement: Supplementary file 1 — Supplementary Information [file 41467_2023_36400_MOESM1_ESM.pdf]

Inventory of Supplementary information

Loss of phosphatase CTDNEP1 potentiates aggressive medulloblastoma by triggering MYC amplification and genomic instability

Zaili Luo, Dazhuan Xin, Yunfei Liao, Kalen Berry, Sean Ogurek, Feng Zhang, Liguo Zhang, Chuntao Zhao, Rohit Rao, Xinran Dong, Hao Li, Jianzhong Yu, Yifeng Lin, Guoying Huang, Lingli Xu, Mei Xin, Ryuichi Nishinakamura, Jiyang Yu, Marcel Kool, Stefan M. Pfister, Martine Roussel, Wenhao Zhou, William A. Weiss, Paul Andreassen, Q. Richard Lu

Contents:

Supplementary Figures 1-17

Supplementary data 1

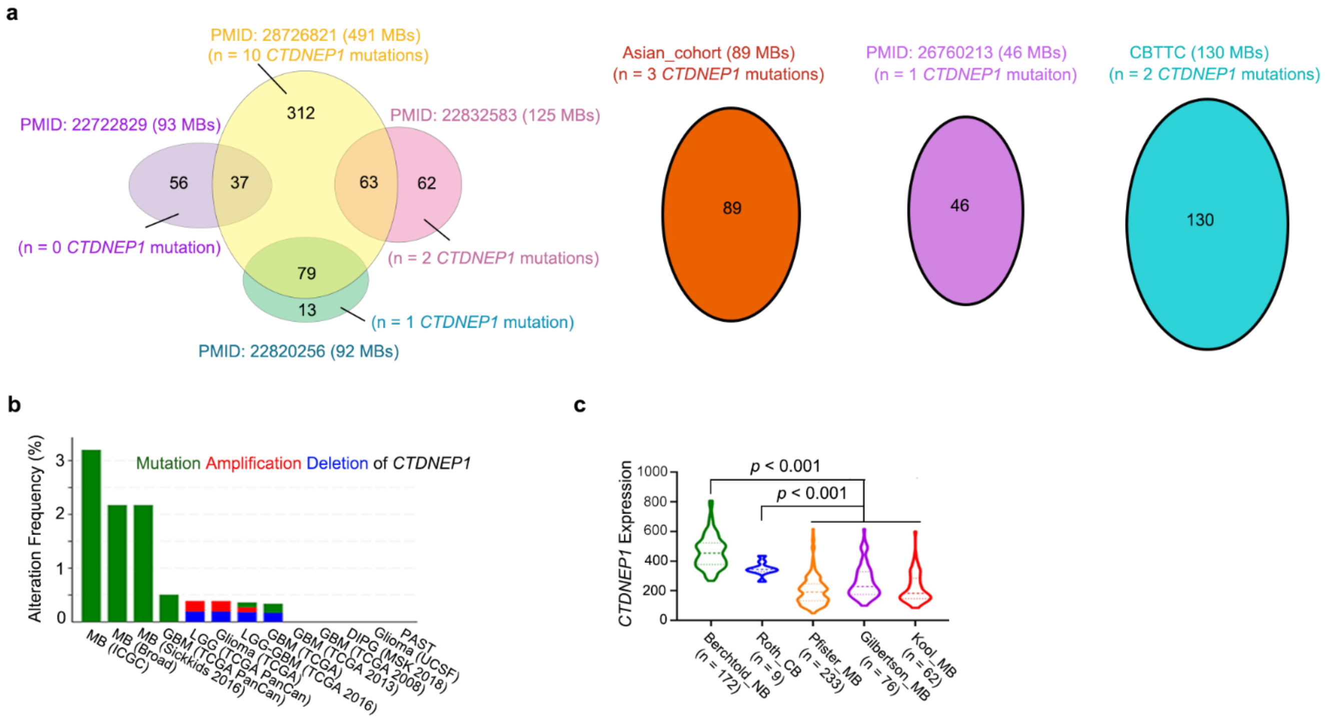

Supplementary Fig.1. The frequency of recurrent mutations across different cohorts.

a, Overview and merge of MB cohorts based on individual patient ID, clinical information, and genomic alterations from WES or WGS profiling show the number of MBs and CTDNEP1 mutation counts in each cohort. **b**, Frequency of alterations in *CTDNEP1* in different types of brain tumors from the TCGA database (www.cbioportal.org). **c**, Analysis of *CTDNEP1* levels in normal brain (NB), cerebella (CB) and MB tumors obtained from indicated MB cohorts. Individual data points with the means ± SD are shown. Two-tailed unpaired Student's *t* test.

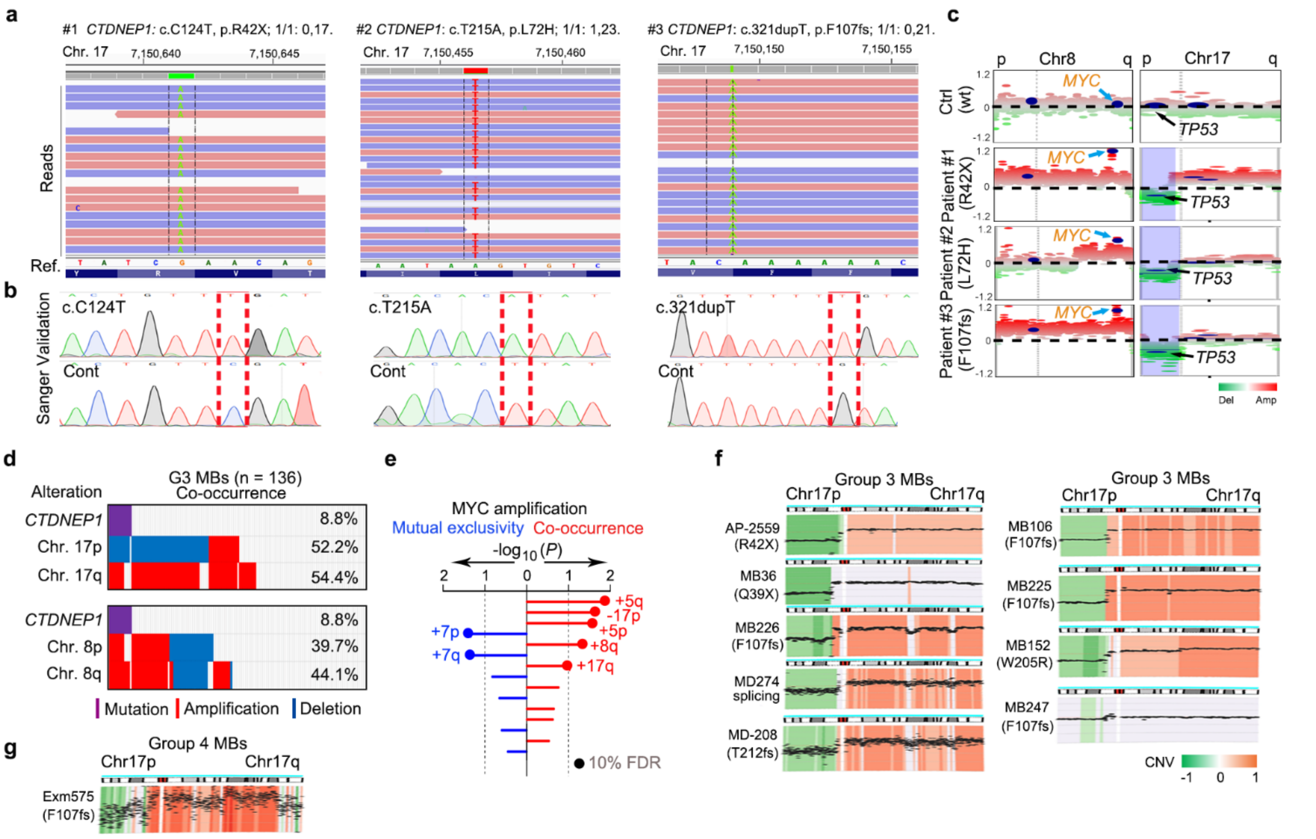

Supplementary Fig. 2. Alterations of nuclear envelope-expressing *CTDNEP1* in medulloblastomas and co-occurrence of genetic alterations with *MYC* amplification.

a, WES sequencing on tumor samples from 89 newly diagnosed patients showing representative G3-MB tumor samples carried LOF *CTDNEP1* mutations, wherein wildtype signals were completely substituted by mutant signals. **b**, Sanger-sequencing validations of homozygous mutations (dash lines) on *CTDNEP1*. **c**, CNV analysis of one peri-tumor brain tissue and three human G3-MBs with *CTDNEP1* mutations based on 850K array methylation, showing LOH for the short arm of chromosome 17p and *MYC* amplification. **d**, Co-occurrence of somatic chromosomal aberrations in *CTDNEP1* mutation-associated G3-MBs. **e**, Co-occurrence between *MYC* amplification and somatic chromosomal alterations, n = 136 G3-MBs. P values were calculated using Bayesian logistic regression analysis, likelihood ratio tests, and adjusted for multiple testing based on FDR correction. **f**,**g**, CNV analysis of Chr17 from nine representative human G3-MB (**f**) and one G4-MB (**g**) with CTDNEP1 mutations from publicly datasets (https://hgserver1.amc.nl/cgi-bin/r2/main.cgi).

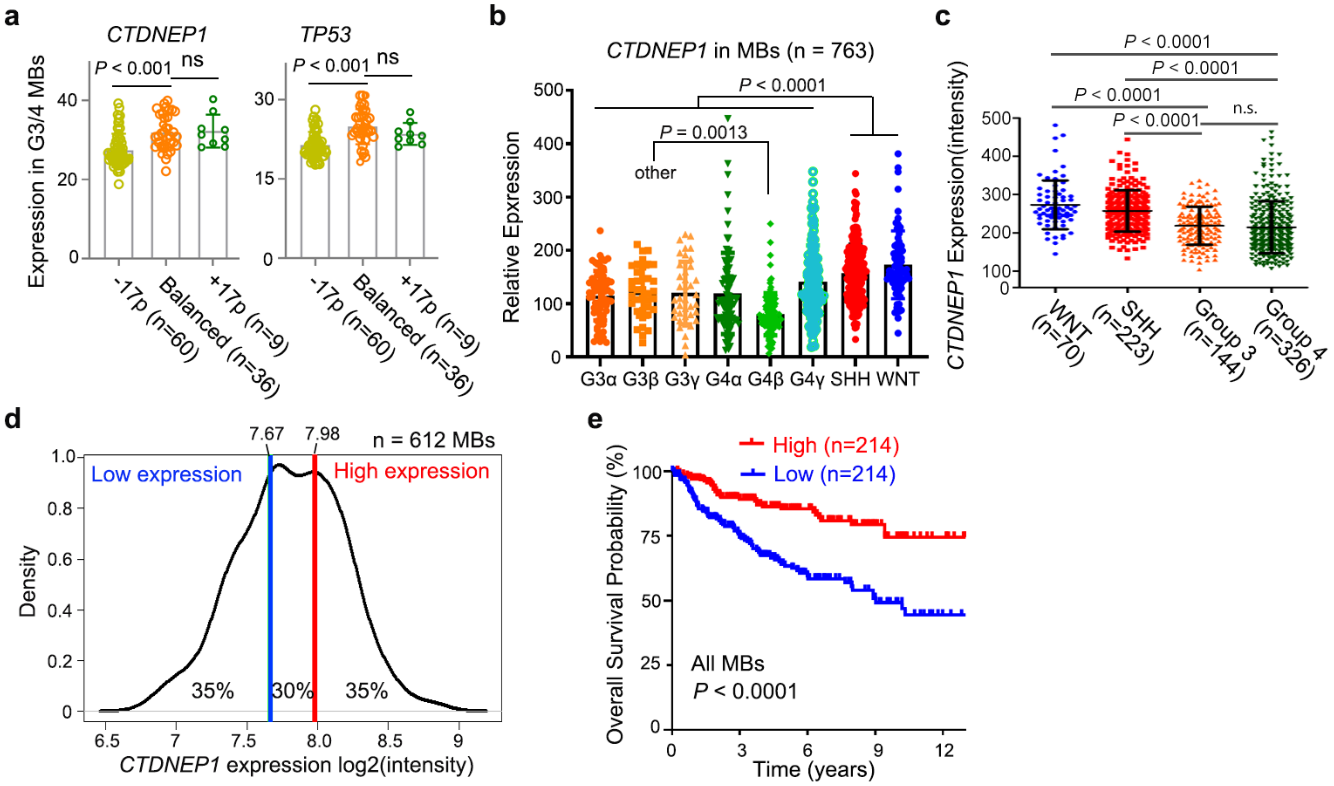

Supplementary Fig. 3. *CTDNEP1* expression levels correlate with MB patient survival across subtypes.

a, *CTDNEP1* and *TP53* expression in G3 and G4 MBs with chromosome 17p loss (n = 63), balanced (n = 38) and 17p gain (n = 5) using the published MB cohort[6]. Data are presented as mean values ± SD; two-tailed unpaired Student's *t*-test. **b**, *CTDNEP1* expression in novel subgroups using publicly available datasets [5]. Data are presented as mean values ± SD, two-tailed unpaired Student's *t*-test. **c**, *CTDNEP1* expression in four subgroups of MBs using publicly available datasets (GEO: gse85217). Data are presented as mean values ± SD, two-tailed unpaired Student's *t*-test. n.s., not significant. **d**, Subgroup of MBs based on population density of *CTDNEP1* expression from 612 MB patients with survival information. Green and red line showed the threshold for 35% low and 35% high expression respectively. **e**, Overall survival of all MB patients with high CTDENP1 expression (35% high) compared with those with low CTDENP1 expression (35% low). *P* value, Log-rank test.

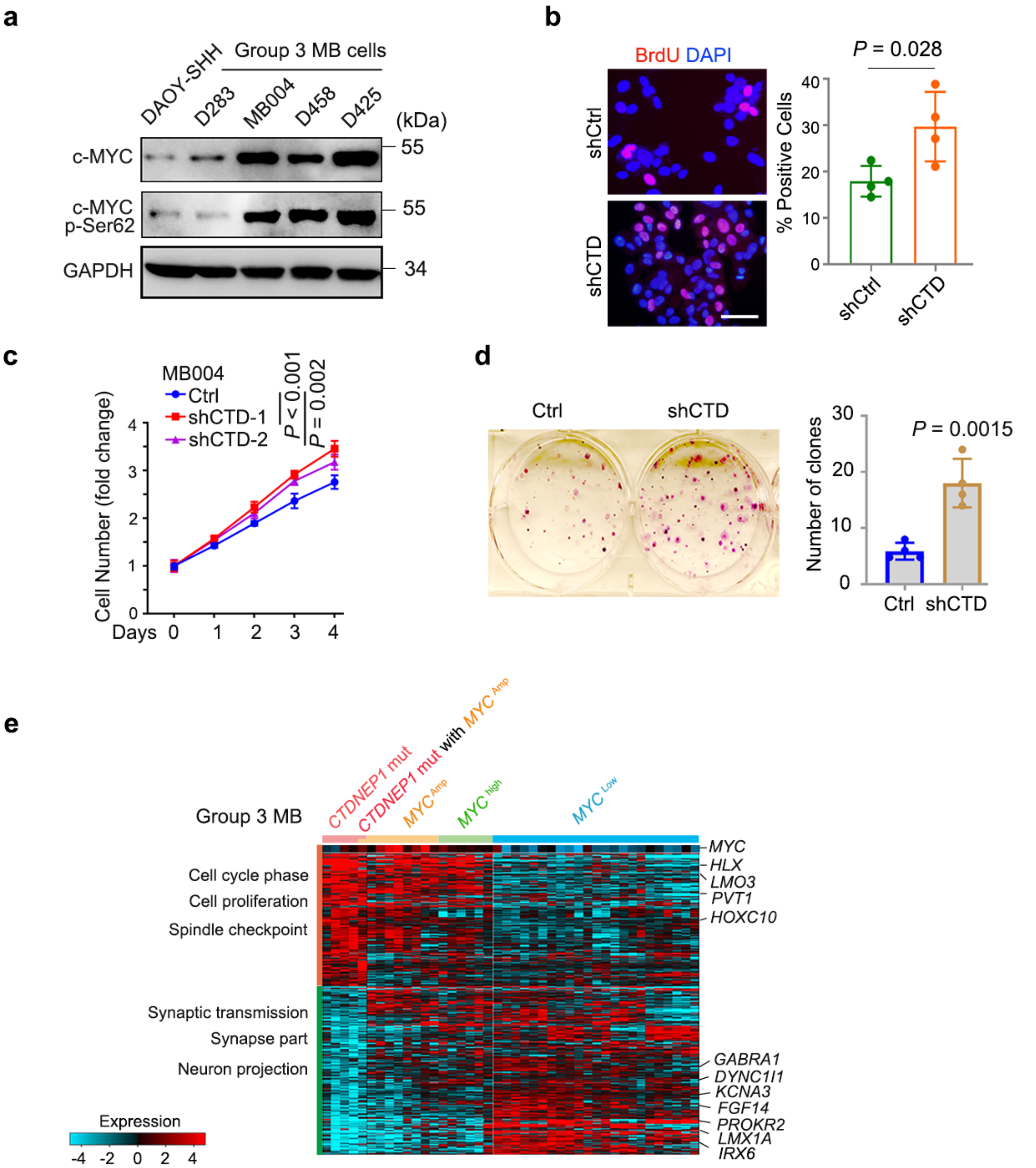

Supplementary Fig. 4. Expression of MYC and MYC target genes regulated by CTDNEP1.
a, MYC and p-Ser62 MYC expression in different human MB cells including MYC amplified G3-MB lines (MB-004, D458 and D425), non-MYC amplified G3 MB, D283, and SHH-MB, DAOY. n = 3 independent experiments. **b**, Left, representative BrdU staining images of G3 MB-004 cells treated with lenti-shRNA control and shCTDNEP1 (scale bar, 50 µm); right, quantification of BrdU $^{+}$ cells. Data represent means ± SD, n = 6 independent experiments. Two-tailed unpaired Student's *t*-test. **c**, Cell proliferation assayed by WST-1 in control and shCTDNEP1 MB-004 cells. Data are presented as mean values ± SD. n = 3 independent experiments, two-tailed unpaired Student's *t*-test. **d**, Left, representative images of colony formation in soft agar assays and right, the number of colonies per well in D425 cells

transduced with shCtrl and shCTDNEP1 for 10 days. Data represent means ± SD, n = 4 independent experiments. Two-tailed unpaired Student's t-test. **e**, Heatmap of differentially expressed genes in published datasets of G3 MBs [6]. *CTDNEP1* mutated (n = 5), 1 *CTDNEP1* mutation with MYC amplification, *MYC* amplification or *MYC* high expression (n = 13), and *MYC* low expression in G3 MB patients (n = 23).

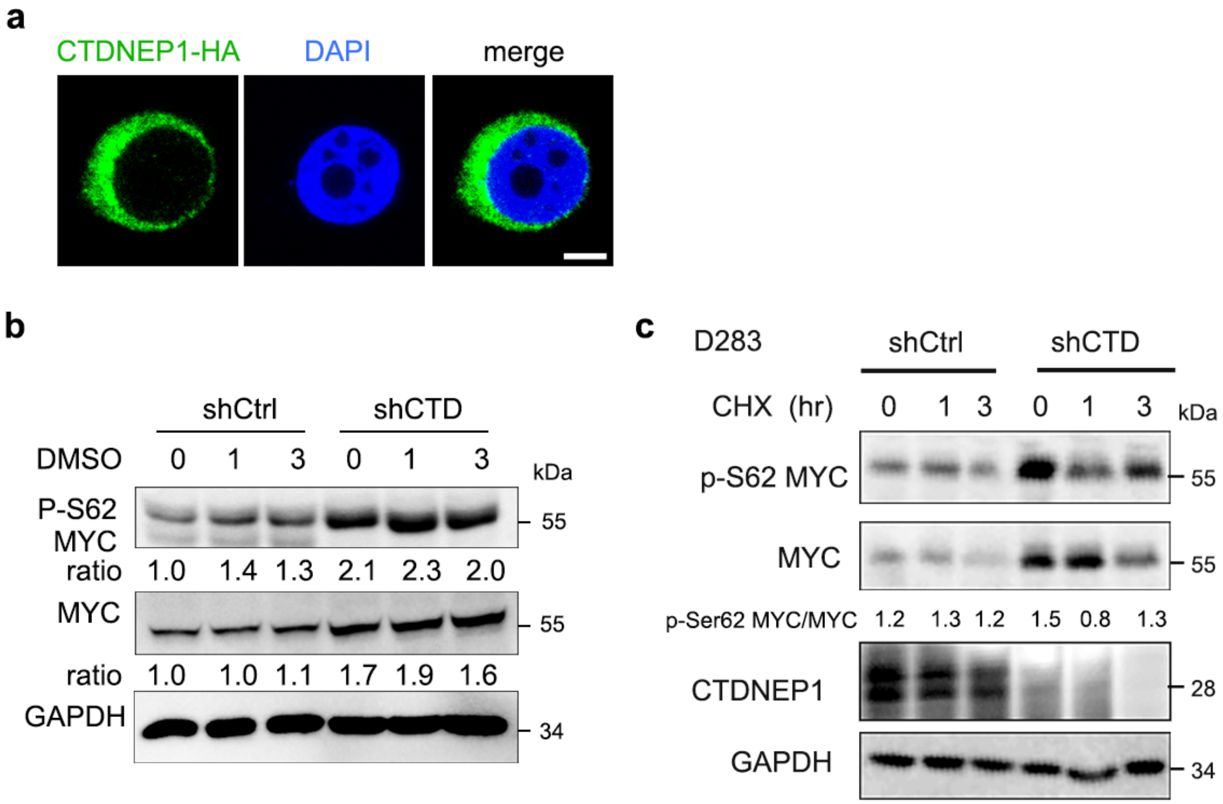

Supplementary Fig. 5. **Deletion of nuclear envelope-enriched CTDNEP1 increases MYC and MYC (p-Ser62) expression**.

a, Representative images showed CTDNEP1 expression enriched around the nuclear membrane in HeLa cells transfected with HA-CTDNEP1. n = 3 independent experiments. Scale bar, 5 µm. **b**, MYC and p-Ser62 expression in D283 cells treated with control and shCTDNEP1 in the presence of cycloheximide (CHX) at the indicated time. n = 3 independent experiments. **c**, Representative immunoblots for total MYC protein and p-S62-MYC in shCtrl and shCTD D425 cells without CHX treatment, showing upregulation of MYC and p-MYC after *Ctdnep1* knockdown (n = 3 independent experiments).

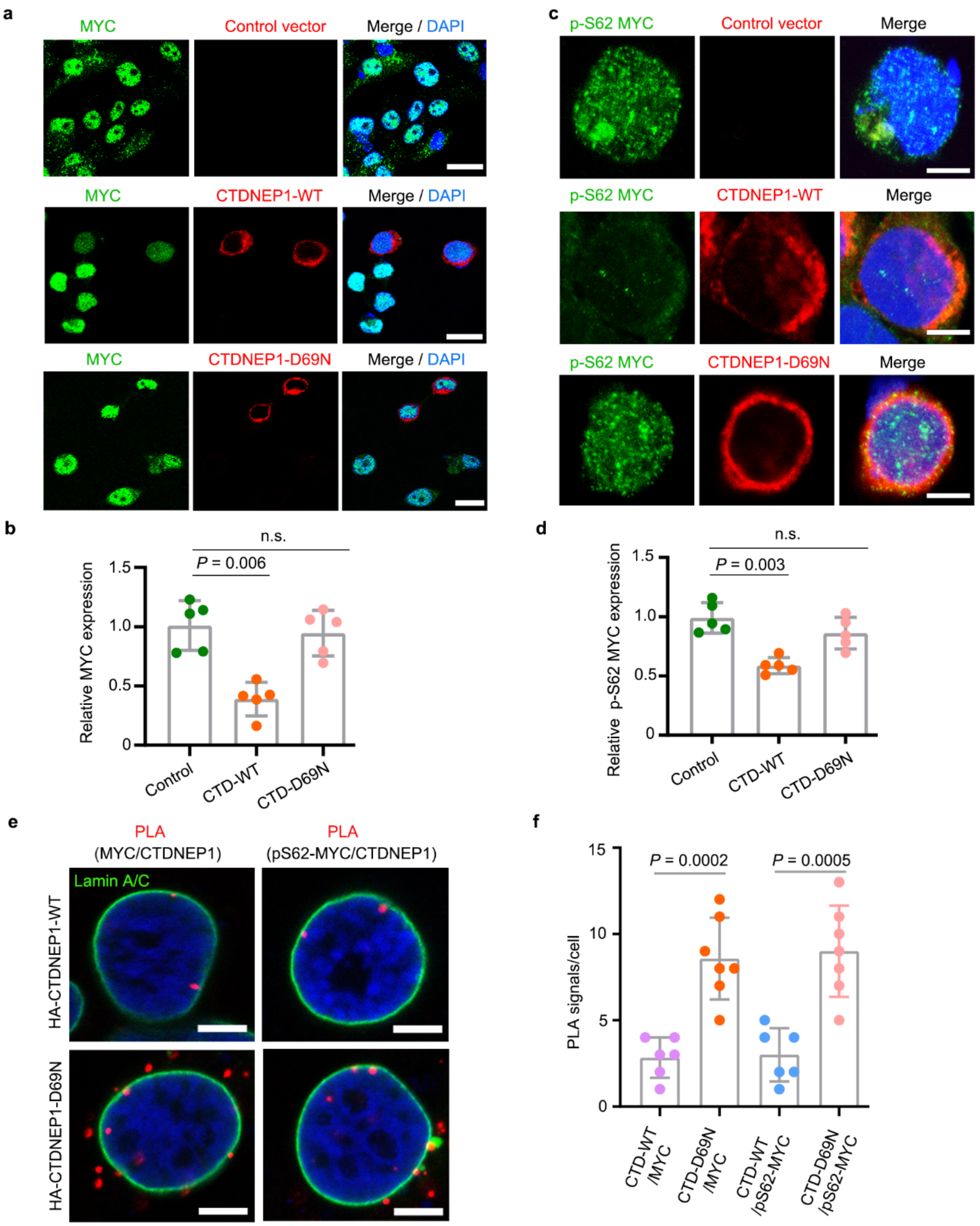

Supplementary Fig. 6. **CTDNEP1 regulates the dynamics of MYC on the nuclear membrane**
a,**b**, Representative images show MYC (green) and HA-tag (red) immunostaining (a) and quantification (b) in HeLa cells transfected with expression vector HA-tagged CTDNEP1-WT or CTDENP1-D69N. **c**,**d**, Representative images (c) and quantification (d) for p62-MYC expression in HeLa cells transfected with the vector expressing HA-CTDNEP1-WT or CTDENP1-D69N. n = 4 independent experiments. n.s., not significant. Two-tailed Student's *t* test. Scale bars in a, 25 μm; c, 5 μm. **e**,**f**, Representative images of co-localization of HA-CTDNEP1(WT or D69N) with MYC or p-S62 MYC with the nuclear

envelope marker lamin A/C (e) and quantification of average number of signals/cell (f) (n = 60 cells) from at least 6 independent transfected experiments in a proximity ligation assay (PLA) in HeLa cells. Two-tailed Student's *t* test. Data are presented as mean values ± SD. Scale bars: 5 μm.

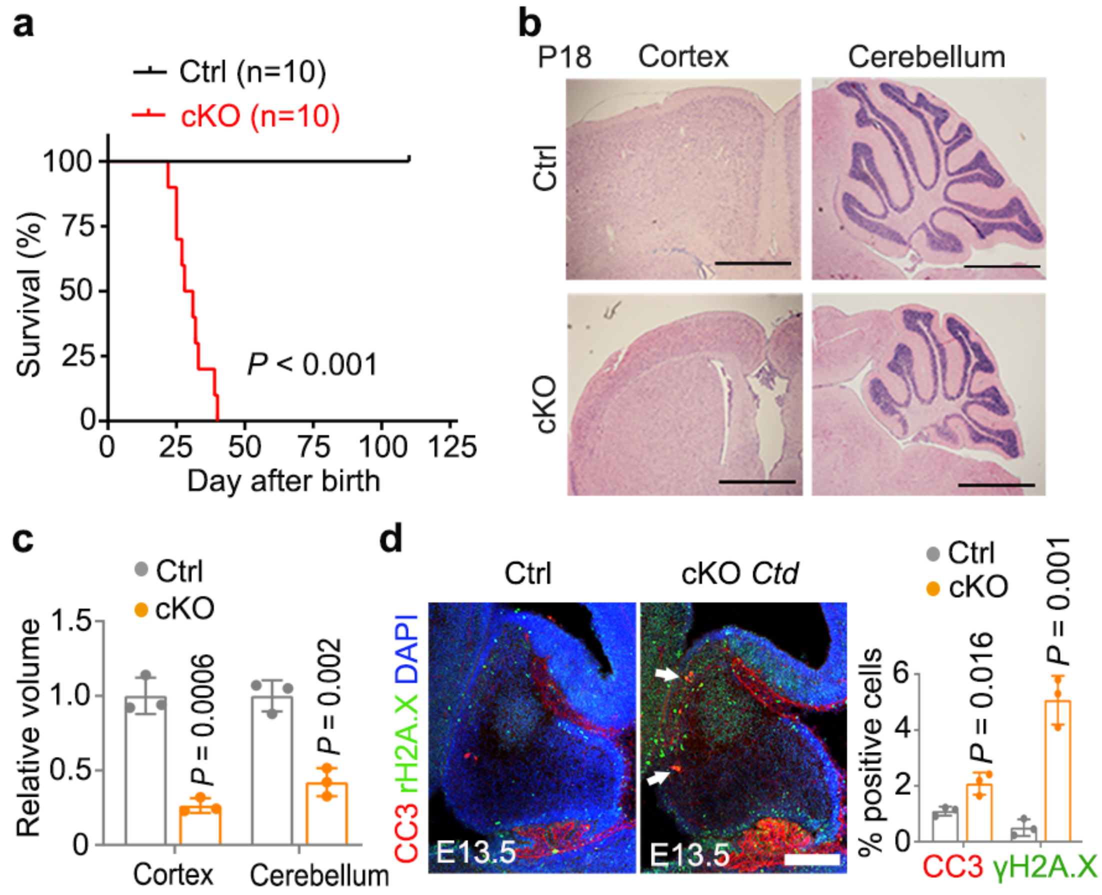

Supplementary Fig. 7. **Deletion of *Ctdnep1* in mouse NPCs induces apoptosis and DNA damage**. **a**, Kaplan-Meier survival curves of mice with Nestin-Cre-mediated knockout of *Ctdnep1*. N = 10 animals per group; $p < 0.001$; log rank test. **b**, Representative images of hematoxylin and eosin stains of the cortex (arrows) and cerebellum from control and *Ctdnep1*-cKO mice at P18. Scale bars: 1 mm. **c**, Bar graph shows the quantification of the relative volume of the cortex and cerebellum from control and *Ctdnep1*-cKO mice at P18. n = 3 animals/genotype. Two-tailed unpaired Student's *t*-test. **d**, Immunofluorescence of cleaved-Caspase 3 and γH2A.X in the control Ctrl and Nestin-Cre *Ctdnep1* cKO- cerebellum at embryo 13.5 (left). Arrows; cleaved-Caspase 3+/γH2A.X+ cells. Scale bars: 200 µm. Right, bar graph shows the quantification of cleaved-Caspase 3 and γH2A.X-positive cells. n = 3 independent measurements. Data are presented as mean values ± SD. Two-tailed unpaired Student's *t*-test.

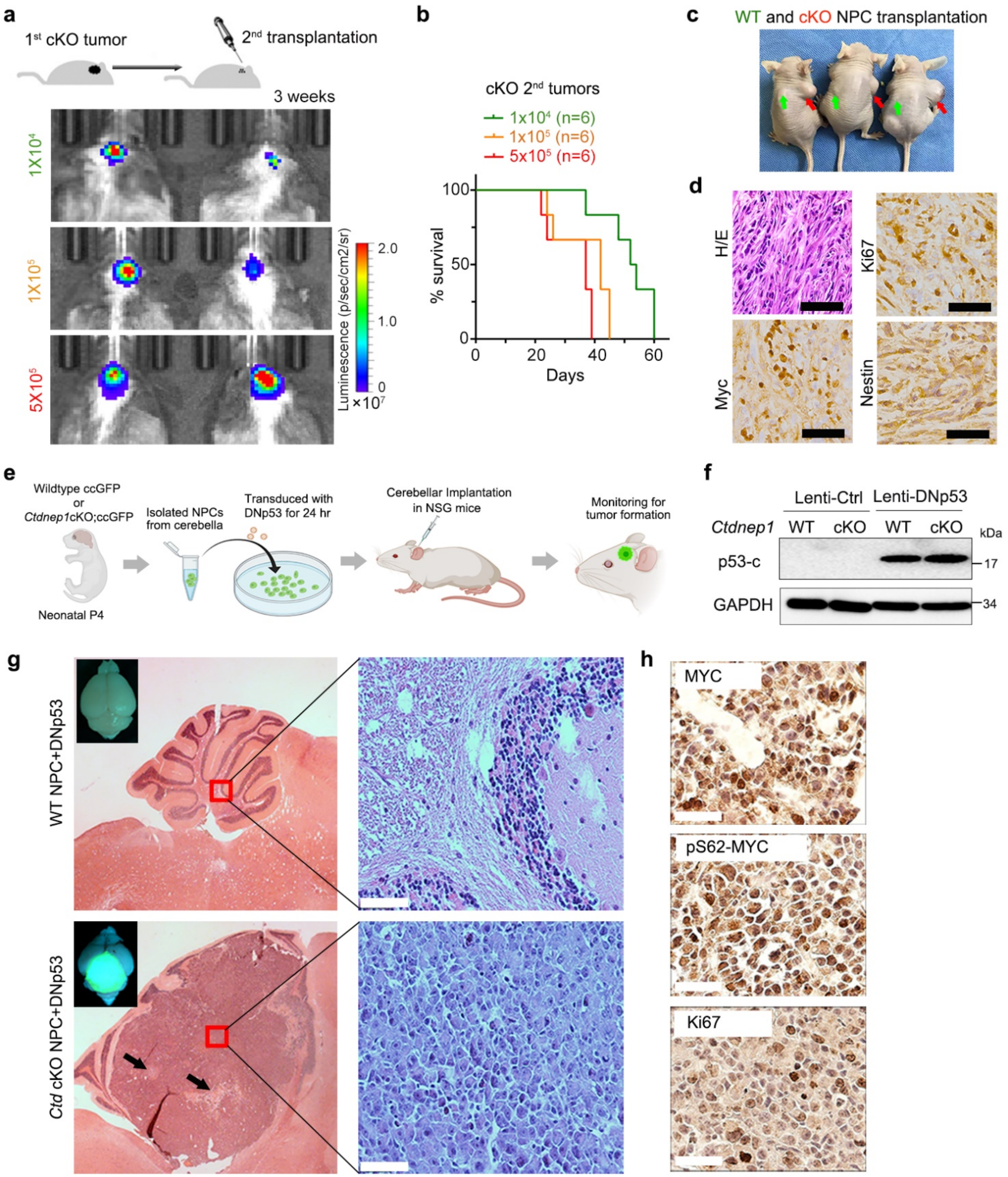

Supplementary Fig. 8. Enhanced tumorigenic potential of *Ctdnep1*-deficient NPCs with p53 inhibition.

a, Upper: Diagram of secondary implantation of tumor cells. Lower: Representative images of bioluminescence imaging of secondary recipients of *Ctdnep1-cKO* NPC tumors. **b**, Survival curves of *Ctdnep1*-cKO tumor cells transplanted into the cerebellum of NSG mice at varying doses. **c**, Tumor

formation in the nude mice subcutaneously transplanted with wildtype (WT) and *Ctdnep1*-cKO (cKO) NPCs (at DIC 15) after 6-month post-transplantation. Green or red arrows show the transplantation sits of WT or cKO NPCs, respectively. **d**, Hematoxylin and eosin staining of subcutaneous *Ctdnep1*-cKO-derived tumors and the representative images of Ki67, c-Myc, and Nestin immunostaining for *Ctdnep1*-cKO-derived tumors. n = 3 independent samples. Scale bars: 50 µm. **e**, Schematic diagram showing that freshly isolated NPCs from *Ctdnep1*-cKO mice at postnatal day 4 were transduced with retroviruses expressing DNp53 for implantation. **f**, Representative western blotting of p53 (c-terminal) expression in NPC cells transduced with lenti-DNp53. n = 3 independent experiments. **g**, Representative G3-MB-like tumor formation in the cerebella transplanted with *Ctdnep1*-cKO NPCs transduced with DNp53 in 6 out of 8 implanted NSG mice. Boxed regions were shown at a high magnification in the right panels. n = 3 independent samples/group. Scale bars: 100 µm. **h**, Immunostaining of MYC, pS62-MYC and Ki67 in the *Ctdnep1*-cKO/DNp53-induced G3-MB-like tumors. n = 3 independent samples. Scale bars: 100 µm.

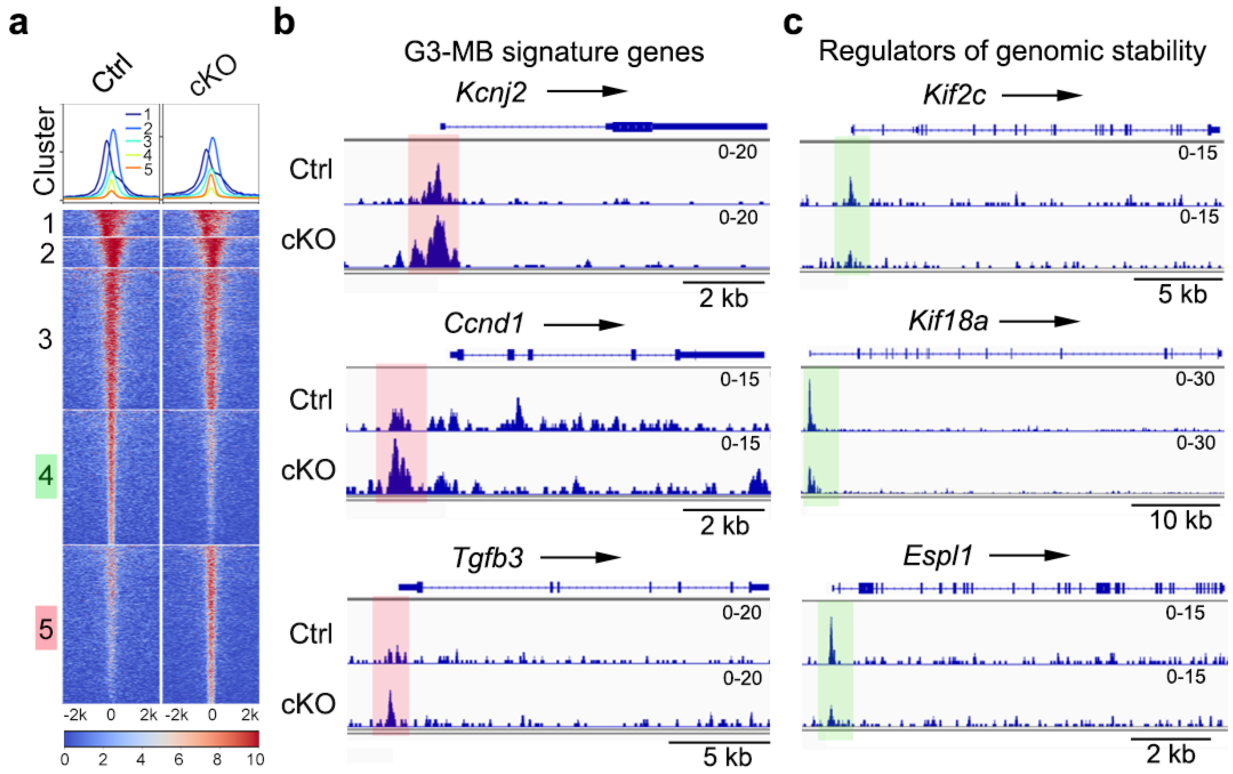

Supplementary Fig. 9. Chromosome access status alteration induced by *Ctdnep1* deletion in mouse NPCs.

a, Heatmaps showing ATAC-seq signals of *Ctdnep1-cKO* and Ctrl NPCs at DIC 10. **b,c**, Representative ATAC-seq signals (highlighted) for G3 MB signature genes (b) and genome stability regulators (c) are shown.

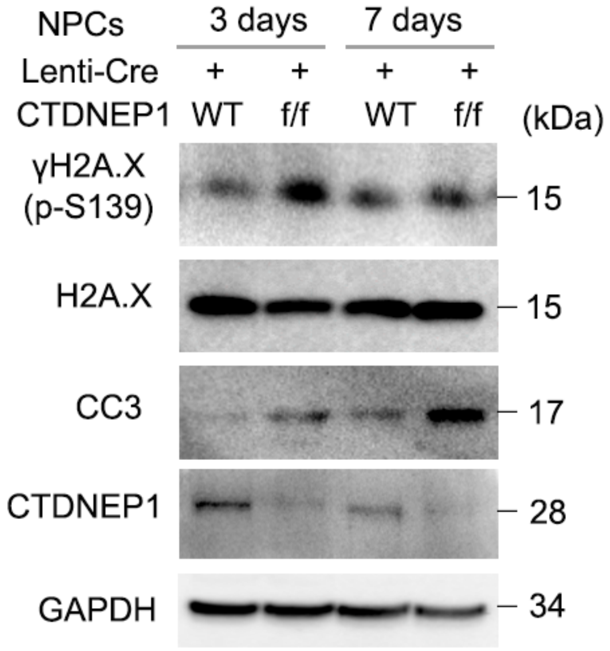

Supplementary Fig. 10. *Ctdnep1* depletion increases DNA damage responses and induces chromosome instability.

Representative immunoblots for gamma H2A.X, total H2A.X, cleaved caspase 3 (CC3), and CTDNEP1 from 3 independent experiments in wildtype or *Ctdenp1*$^{fl/fl}$ NPCs transduced with lenti-Cre at day 3 and day 7.

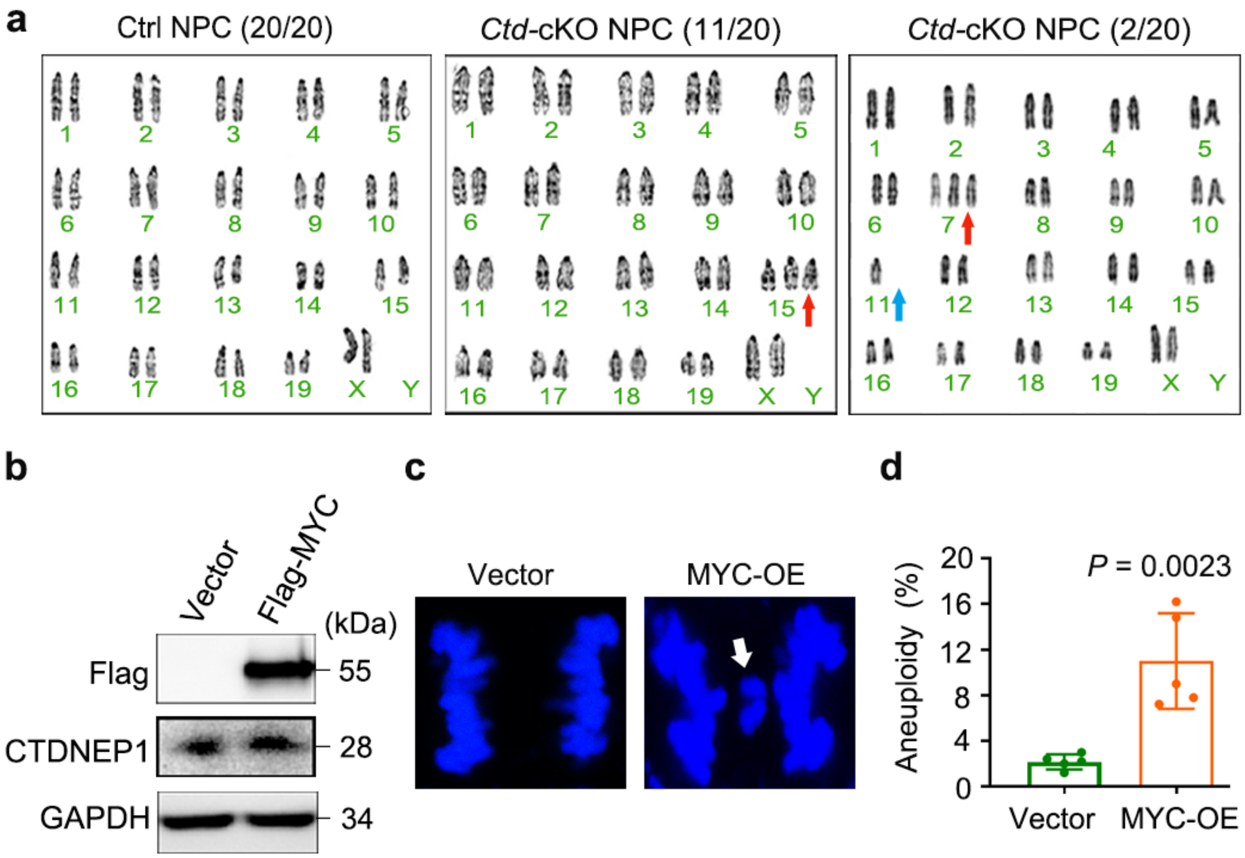

Supplementary Fig. 11. **Karyotypes of *Ctdnep1*-cKO NPCs and the chromosome instability induced by MYC overexpression.**

a, Karyotypes of control and *Ctdnep1*-cKO NPCs at interim-stage (DIC 45) scored from 20 spreads each. Red arrow points to the triplicated chromosome 15. **b**, Representative immunoblotting for MYC and CTDNEP1 expression in D283 cells transduced with control and lenti-Flag-MYC. n = 3 independent experiments. **c**, **d**, Representative image (c) and quantification (d) of abnormal anaphases in the cells with or without MYC overexpression. Arrow: lagging chromosomes, n = 5 independent experiments. Data are presented as mean values ± SD. Two-sided Student's *t* test.

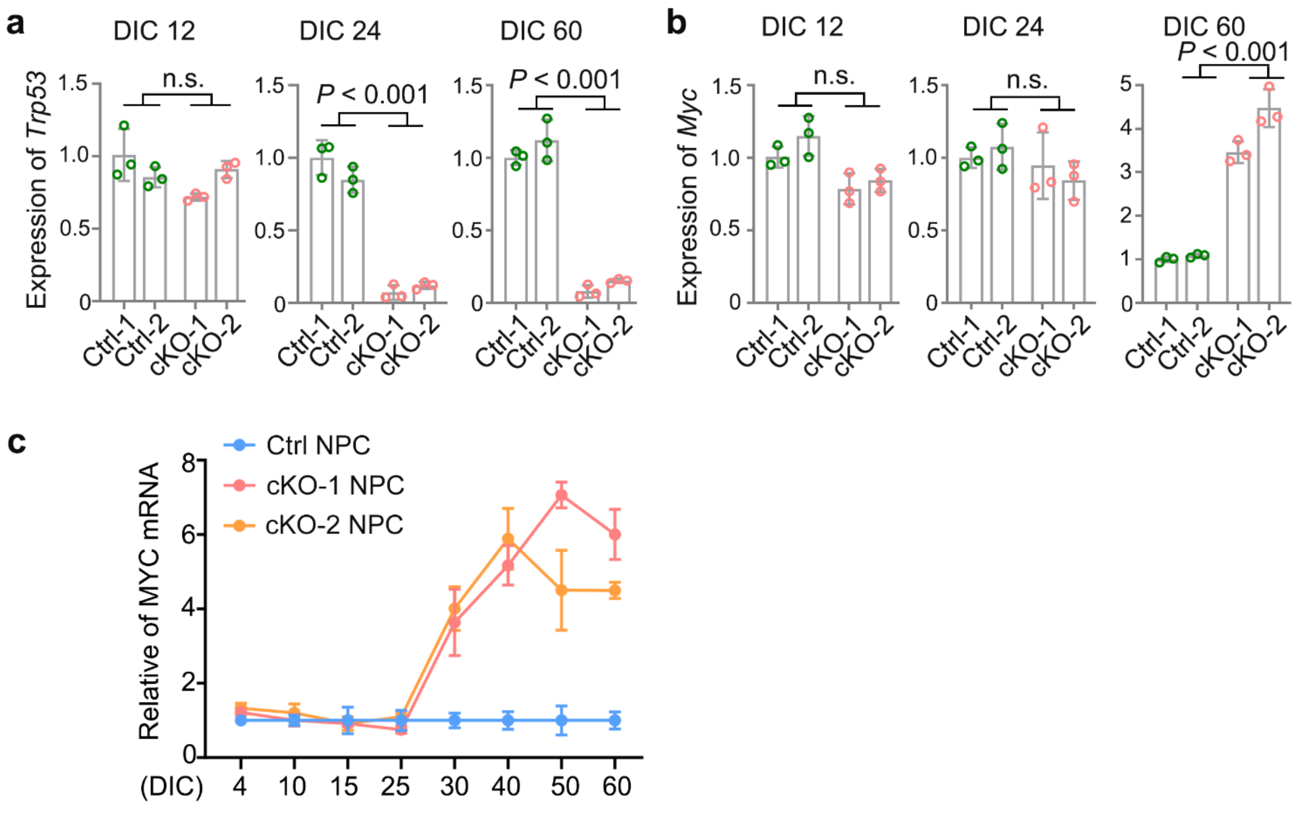

Supplementary Fig. 12. **mRNA of *Trp53* and *Myc* expression in the cKO-*Ctdnep1* NPCs.**
a and **b**, mRNA of *Trp53* (**a**) and *Myc* (**b**) expression in cKO-*Ctdnep1* NPCs (cKO-1 and cKO-2) and control NPCs (Ctrl-1 and Ctrl-2) at the early-stage (DIC 12), interim-stage (DIC 24) and late-stage (DIC 60). n.s., not significant, n = 3 independent experiments, two-tailed Student's *t*-test. **c**, *Myc* mRNA expression in control and cKO-*Ctdnep1* NPCs at the indicated time points in culture, n = 3 independent experiments. Data are presented as mean values ± SD.

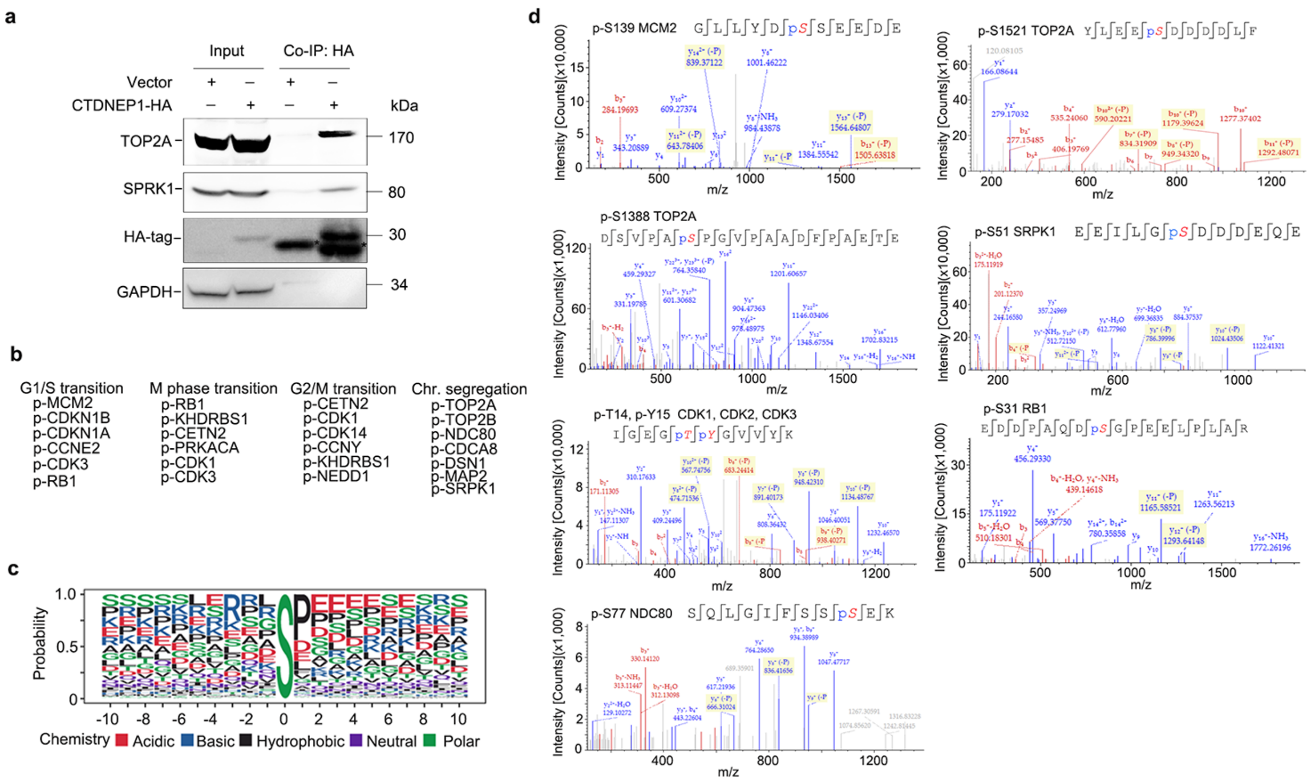

Supplementary Fig. 13. **Validation of CTDNEP1 binding proteins and identification of phospho-proteins upregulated in *Ctdnep1*-cKO NPCs.**

a, Representative immunoblots (n = 3 independent experiments) for CTDNEP1 binding with TOP2A and SPRK1 in D425 cells transduced with control and lentivirus expressing HA-tag-CTDNEP1. Asterisks indicate the light chain of IgG. **b**, List of the cell-cycle related phosphorylated proteins that are enriched in *Ctdnep1*-cKO NPCs compared to wild-type NPCs. **c**, Motif analysis of phosphorylated proteins based on the phosphorylation site sequences enriched in *Ctdnep1* cKO NPCs compared with control NPCs. **d**, Mass spectrometry identification of phospho-proteins that are upregulated in *Ctdnep1*-cKO NPCs.

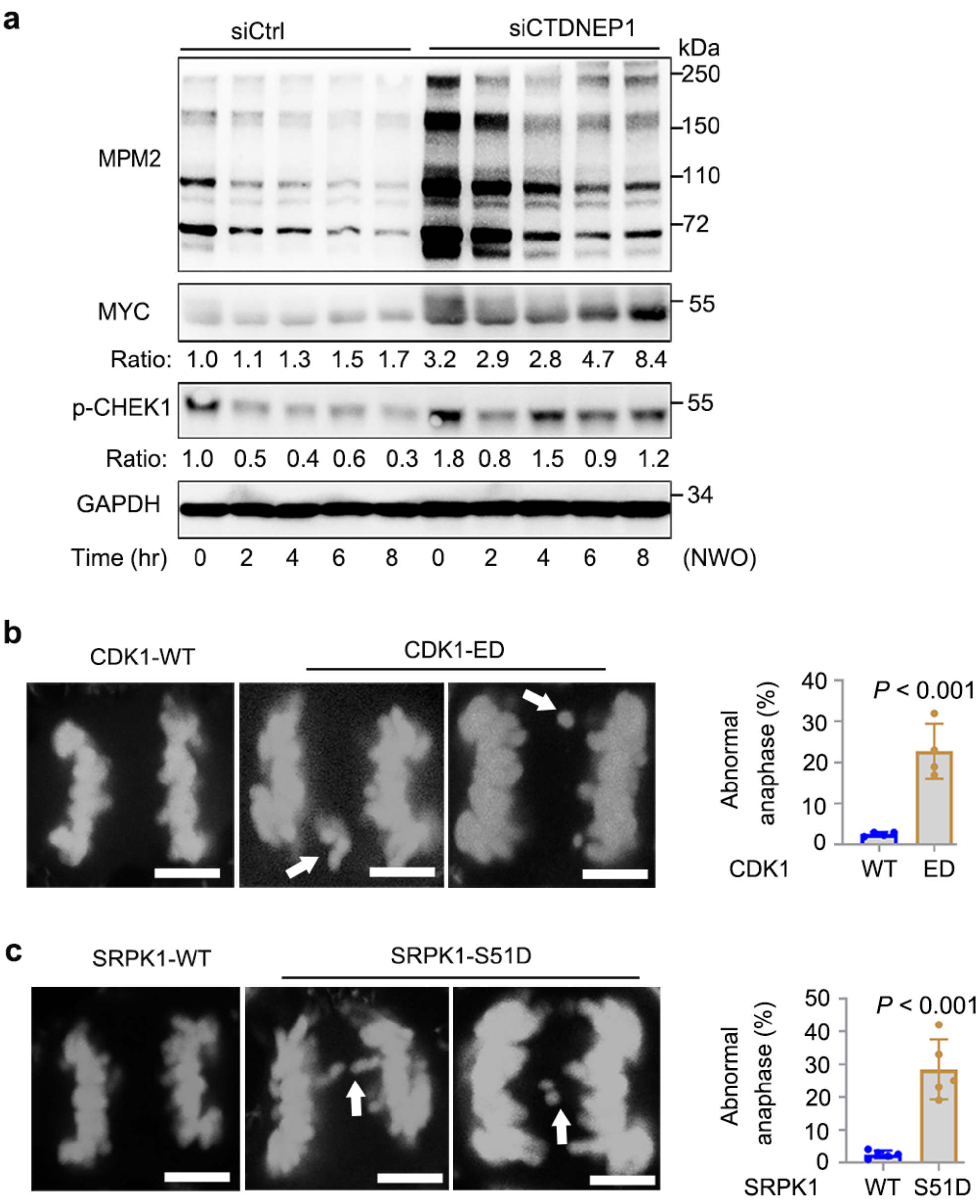

Supplementary Fig. 14. **Inhibition of CTDNEP1 activates the mitotic check points and chromosomal abnormalities induced by the CTDNEP1 potential candidates.**

a, Representative immunoblots from 3 independent experiments for the indicated phospho-proteins in DAOY cells transfected with control siRNA or siCTDNEP1 after treatment with nocodazole for 14 h and sampled at indicated timepoints in fresh medium. NOW; nocodazole washout. **b**,**c**, Representative images (left) and quantification (right) of the average frequencies of chromosomal abnormalities in the U2OS cells transfected with control vector, CDK1-WT or CDK1-ED (b) and SRPK1-WT and SRPK1-S51D forms (c), respectively. n = 3 independent experiments. Anaphase cells (at least 60 cells/group) were counted. Data are presented as mean values ± SD. Two-tailed unpaired Student's *t*-test. Scale bars, 5 μm.

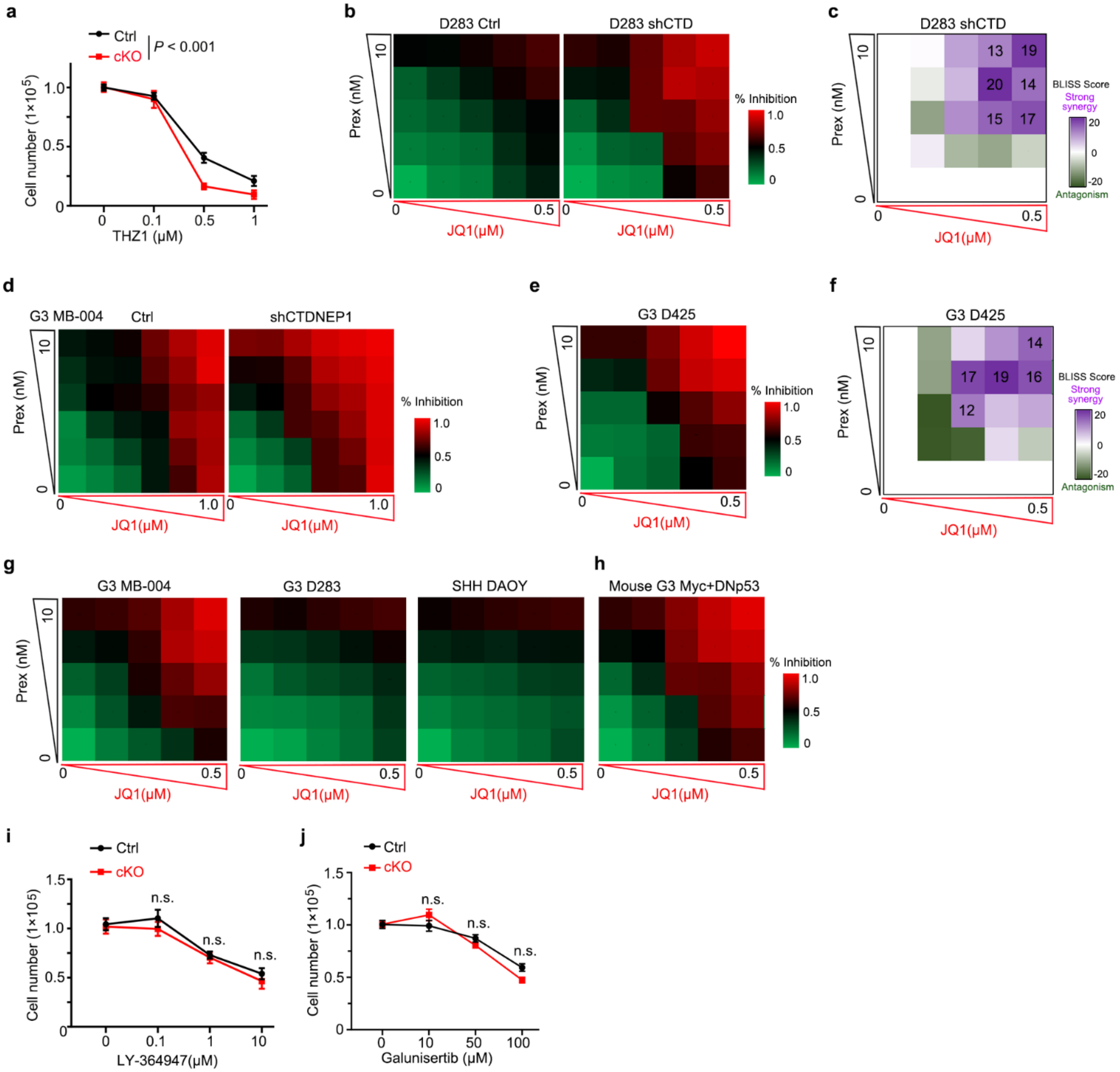

Supplementary Fig. 15. **MB cell proliferation is inhibited by small-molecule inhibitors of c-MYC and CHEK1, but not TGF-β signaling.**

a, Cell viability of control and *Ctdnep1*-cKO NPCs treated with the indicated concentrations of THZ1 relative to vehicle-treated cells. Data represent means ± SD, n = 5 independent experiments. Two-way ANOVA. **b,c**, Heatmap showing the percentage of growth inhibition of control and shCTD-treated D283 cells by combined treatment with JQ1 and prexasertib relative to vehicle-treated cells (b); Bliss score for JQ1 and prexasertib double titrations (c). n = 5 independent experiments. **d**, Heatmap showing the percentage of growth inhibition in MB-004 cells transduced with shCtrl and shCTDNEP1 after combined JQ1 and prexasertib treatment relative to vehicle-treated cells. n = 5 independent experiments. **e,f**, Heatmap showing the percentage of growth inhibition of D425 cells with combined treatment of JQ1 and prexasertib relative to vehicle-treated cells. n = 5 independent experiments (**e**), Bliss score for treatment with JQ1 and prexasertib double titrations (f). **g,h**, Heatmap showing the percentage of the

growth inhibition of MYC amplified human G3 MB-004 and mouse G3 (MYC+DNp53) tumor cells (g), and non-MYC amplified cells (G3 MB D283 and SHH-MB DAOY cells (h) treated with combined treatment of JQ1 and prexasertib relative to vehicle-treated cells. n = 5 independent experiments. **i,j**, Cell viability of control and *Ctdnep1*-cKO NPC tumor cells treated with TGFβ pathway inhibitors LY-364947 (i) or galunisertib (j) relative to vehicle-treated controls. n = 5 independent experiments. Data are presented as mean values ± SD. n.s., no significance, two-tailed unpaired Student's *t*-test.

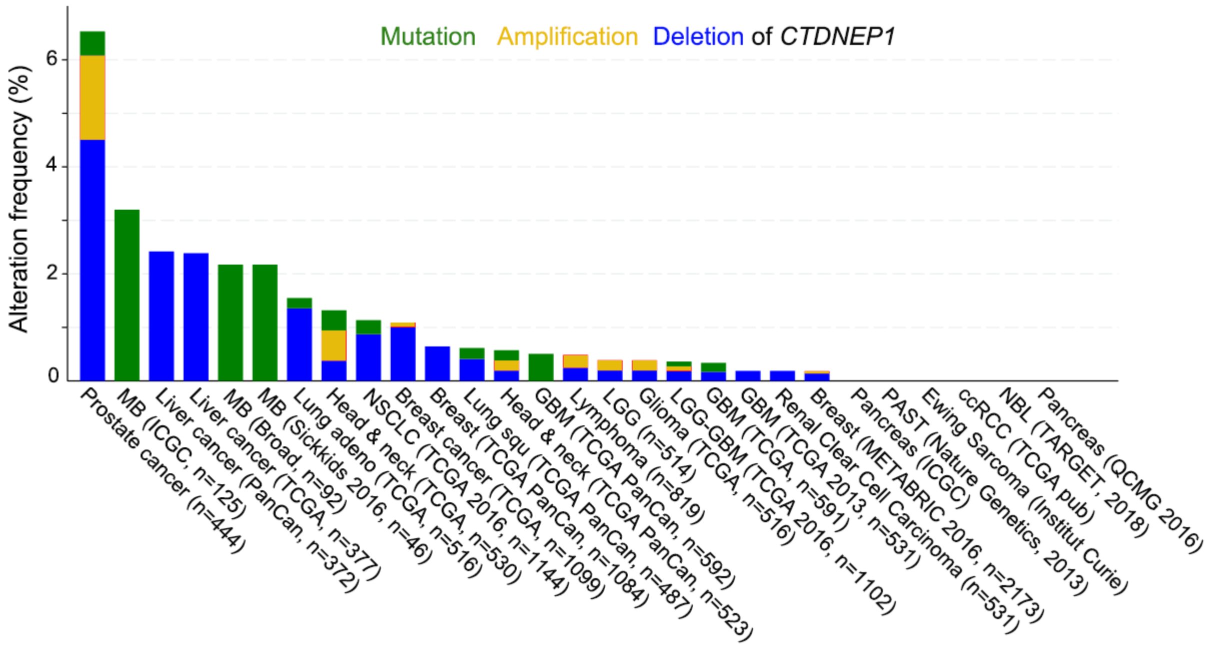

Supplementary Fig. 16. **Alterations of *CTDNEP1* in patient samples.**

Frequency of alterations in *CTDNEP1* in different tumor types from the TCGA database (www.cbioportal.org).

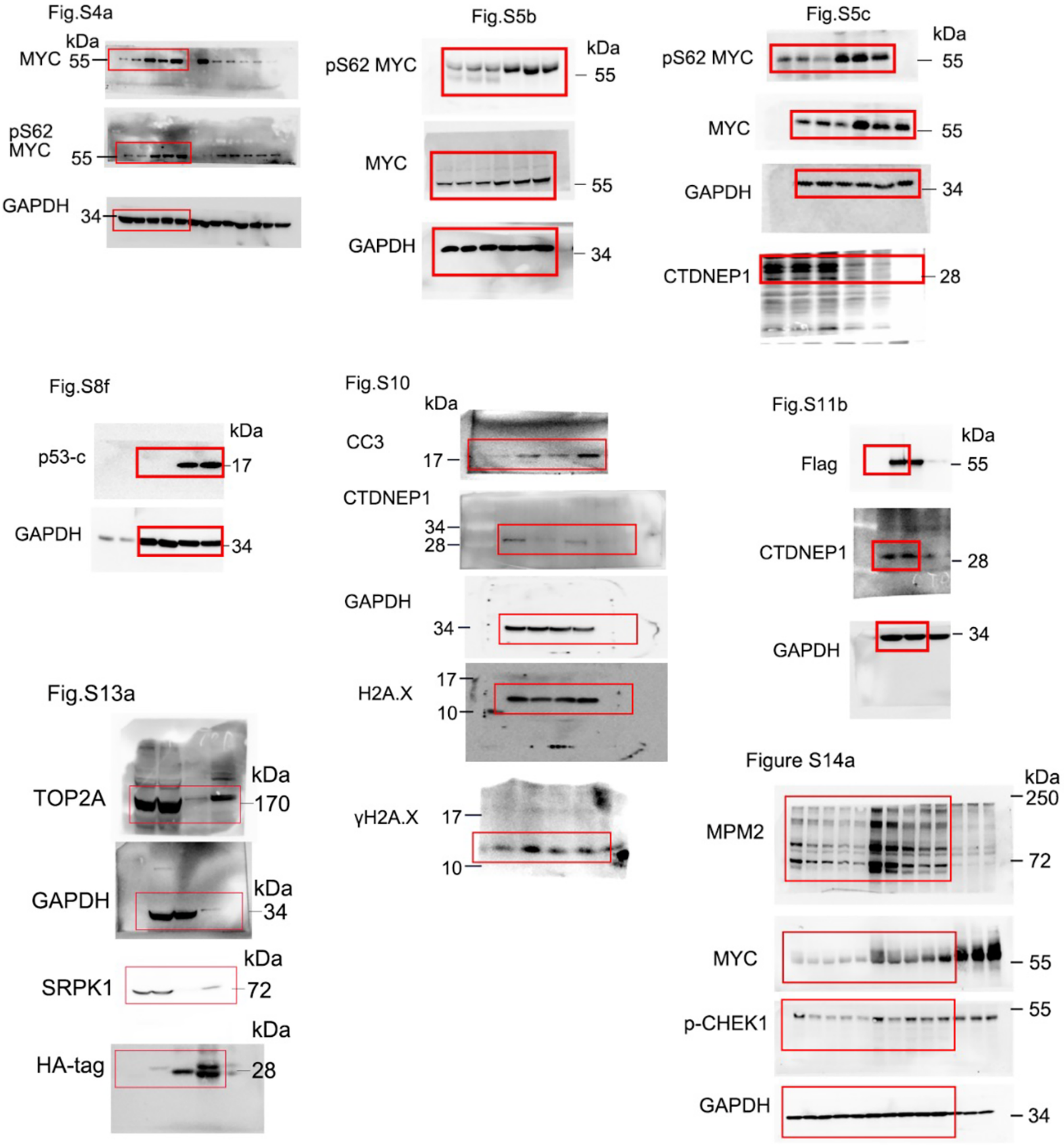

Supplementary Fig.17. Full blots for Supplementary Figures 4, 5, 8, 10, 11, 13, 14.
