# Supplementary material for: Loss of phosphatase CTDNEP1 potentiates aggressive medulloblastoma by triggering MYC amplification and genomic instability
Source: Nat Commun. 2023 Feb 10;14:762. doi: 10.1038/s41467-023-36400-8 (PMC9918503; doi:10.1038/s41467-023-36400-8)
Supplement: Supplementary file 2 — Description of Additional Supplementary Files [file 41467_2023_36400_MOESM2_ESM.pdf]

**Description of Additional Supplementary Files**

File Name: Supplementary Data 1
Description: Sequence of primers for the QPCR and shRNA
