# Supplementary material for: Loss of phosphatase CTDNEP1 potentiates aggressive medulloblastoma by triggering MYC amplification and genomic instability
Source: Nat Commun. 2023 Feb 10;14:762. doi: 10.1038/s41467-023-36400-8 (PMC9918503; doi:10.1038/s41467-023-36400-8)
Supplement: Supplementary file 4 — Reporting Summary [file 41467_2023_36400_MOESM4_ESM.pdf]

nature portfolio

Corresponding author(s): Q. Richard Lu

Last updated by author(s): Dec 28, 2022

Reporting Summary

Nature Portfolio wishes to improve the reproducibility of the work that we publish. This form provides structure for consistency and transparency in reporting. For further information on Nature Portfolio policies, see our Editorial Policies and the Editorial Policy Checklist.

Statistics

For all statistical analyses, confirm that the following items are present in the figure legend, table legend, main text, or Methods section.

n/a	Confirmed	
☐	☒	The exact sample size (*n*) for each experimental group/condition, given as a discrete number and unit of measurement
☐	☒	A statement on whether measurements were taken from distinct samples or whether the same sample was measured repeatedly
☐	☒	The statistical test(s) used AND whether they are one- or two-sided<br>*Only common tests should be described solely by name; describe more complex techniques in the Methods section.*
☐	☒	A description of all covariates tested
☐	☒	A description of any assumptions or corrections, such as tests of normality and adjustment for multiple comparisons
☐	☒	A full description of the statistical parameters including central tendency (e.g. means) or other basic estimates (e.g. regression coefficient) AND variation (e.g. standard deviation) or associated estimates of uncertainty (e.g. confidence intervals)
☒	☐	For null hypothesis testing, the test statistic (e.g. *F*, *t*, *r*) with confidence intervals, effect sizes, degrees of freedom and *P* value noted<br>*Give P values as exact values whenever suitable.*
☒	☐	For Bayesian analysis, information on the choice of priors and Markov chain Monte Carlo settings
☒	☐	For hierarchical and complex designs, identification of the appropriate level for tests and full reporting of outcomes
☐	☒	Estimates of effect sizes (e.g. Cohen's *d*, Pearson's *r*), indicating how they were calculated

Our web collection on statistics for biologists contains articles on many of the points above.

Software and code

Policy information about availability of computer code

Data collection	Nikon C2 plus confocal for imaging. RNA-seq libraries were prepared using Illumina RNA-Seq Preparation Kit and sequenced by a HiSeq 2500 sequencer.<br>ATAC-seq libraries were purified with AMPure beads (Agencourt) to remove contaminating primer dimers and were sequenced on the Illumina HiSeq 2500 with 75 bp single-end reads.<br>Whole genome sequencing (WGS) libraries were prepared using WGS Preparation Kit and sequenced by DNBseq™ sequencing technology.
Data analysis	Generally, most of the data are processed using the following softwares: GraphPad Prism 8, Thermo Proteome Discoverer (2.3.0.523), AltAnalyze (v.2.1.0, http://www.altanalyze.org/), GSEA (Win_4.0.3), FlowJo (v10), DESeq2(v1.22.2), RStudio (v.3.4.0), Enrichr (https://maayanlab.cloud/Enrichr/ for functional enrichment analysis), Galaxy (https://usegalaxy.org/), Toppgene (https://toppgene.cchmc.org/ for gene functional category enrichment analysis).<br><br>RNA-seq: Reads mapped to reference genome mm10 (https://hgdownload.soe.ucsc.edu/downloads.html) by Tophat (http://tophat.cbcb.umd.edu). We used Cuff-diff and Bioconductor (v.1.4.0) DESeq package to identify differentially expressed genes. Gene ontology (GO) analysis was performed using ToppGene Suite (https://toppgene.cchmc.org/) and Gene Set Enrichment Analysis (GSEA; http://www.broadinstitute.org/gsea/index.jsp).<br><br>WGS CNV analysis: Data were mapped to the mm10 reference genome with the ISAAC aligner. The resulting BAM files were analyzed with version 1.3.9 of the Canvas CNV caller. CNVs were called using SOAPcnv software. Based on the result of SOAP alignment, the depth of each base should be calculated and standardized by the mean depth of its chromosome to calculated the copy number variation. CNVs were detected by the following steps: 1) DNA sequences were separated into fragments according to the depth of each base from the alignment results; 2) The P-value was calculated for each fragment to estimate its probability to be a CNV ; 3) The fragments that passed the criteria

(fragment length longer than 2 kb, P-value <= 0.35, mean depth less than 0.5 or more than 2.0) were kept as CNVs. The mapped bam files from WGS were used for CNV analysis. We followed the somatic copy number variation pipeline from GATK4 CNV (https://github.com/ding-lab/gatk4wxscnv). The final segment ratio files with CNV type annotation for all NPC and tumor samples were further annotated by AnnotSV.
ATAC-seq: Reads mapped to rn5 whole genome by Bowtie2 with default options. Peak calling was performed using Model-based analysis of MACS version v2.12 with special parameter: --shift -75 --extsize 150 --nomodel --call-summits --nolambda --keep-dup all -p 0.01, to call peak, which extend and shift the fragments to get the region cut by the Tn5 sites.

For manuscripts utilizing custom algorithms or software that are central to the research but not yet described in published literature, software must be made available to editors and reviewers. We strongly encourage code deposition in a community repository (e.g. GitHub). See the Nature Portfolio guidelines for submitting code & software for further information.

Data

Policy information about availability of data

All manuscripts must include a data availability statement. This statement should provide the following information, where applicable:

- Accession codes, unique identifiers, or web links for publicly available datasets
- A description of any restrictions on data availability
- For clinical datasets or third party data, please ensure that the statement adheres to our policy

All high-throughput data generated in the paper are deposited in the NCBI Gene Expression Omnibus (GEO). The accession number is GSE145921. The mass spectrometry proteomics datasets are deposited in ProteomeXchange (Identifier: PXD019067). Source data are provided with this paper.

Human research participants

Policy information about studies involving human research participants and Sex and Gender in Research.

Reporting on sex and gender	Sex and gender information of patients is reported in source data.
Population characteristics	Population characteristics were not used in this study.
Recruitment	Participants were recruited based on their being treated on hospital protocols. No recruitment criteria other than consent was required.
Ethics oversight	The use of tumor samples was approved by individual institutional review boards (IRB) from Cincinnati Children's Hospital and Children's Hospital of Fudan University. Informed consents for the use of tissues for research were obtained in writing from donors or the patients' parents.

Note that full information on the approval of the study protocol must also be provided in the manuscript.

Field-specific reporting

Please select the one below that is the best fit for your research. If you are not sure, read the appropriate sections before making your selection.

☒ Life sciences ☐ Behavioural & social sciences ☐ Ecological, evolutionary & environmental sciences

For a reference copy of the document with all sections, see nature.com/documents/nr-reporting-summary-flat.pdf

Life sciences study design

All studies must disclose on these points even when the disclosure is negative.

Sample size	Sample sizes were indicated in the legend of each Figure and Supplementary Figure. The sample size was chosen empirically, based on common experience in the relevant fields, to provide a sufficient level of statistical power for detecting indicated biological effects. No statistical methods were used to pre-determine the sample size.
Data exclusions	No data are excluded from this study.
Replication	The experimental findings were reliably reproduced. In vitro experiments were performed with two or more replicates to capture variability. All replication attempts were successful. For in vivo animal experiments, biological replicates were carried out using independent animals. The number of replicates was described in the figure legends and statistics section.
Randomization	The mice were randomly assigned (with both sex) into different groups for drug treatment experiments. There was no allocation of test subjects for any other experiments, thus randomization is not relevant beyond animal experiments in this study.
Blinding	Sample blinding was not performed because the knowledge of experimental conditions was required during data collection and analyses. Cell lines/tissues were known before the conduction of experiments, but data were quantified with blinding. No hypothesis was tested regarding molecular identities. All samples were treated equally using the same rigorous criteria to avoid bias.

Reporting for specific materials, systems and methods

We require information from authors about some types of materials, experimental systems and methods used in many studies. Here, indicate whether each material, system or method listed is relevant to your study. If you are not sure if a list item applies to your research, read the appropriate section before selecting a response.

Materials & experimental systems

n/a	Involved in the study
☐	☒ Antibodies
☐	☒ Eukaryotic cell lines
☒	☐ Palaeontology and archaeology
☐	☒ Animals and other organisms
☒	☐ Clinical data
☒	☐ Dual use research of concern

Methods

n/a	Involved in the study
☒	☐ ChIP-seq
☐	☒ Flow cytometry
☒	☐ MRI-based neuroimaging

Antibodies

Antibodies used

For western blot experiments, we used antibodies against Cleaved Caspase 3 (Rabbit, Cell Signaling, Cat# 9661, 1:1000), c-Myc (Mouse, Cell Signaling; Cat#5605S, 1:1000), γH2A.X (Rabbit, Cell Signaling, Cat# 9718S, 1:1000), p53 (Mouse, Cell Signaling, Cat# 2524S, 1:1000), MPM2 (Mouse, Millipore, Cat# 05-368, 1:1000), p-S317 Chk1(Rabbit, Cell Signaling, Cat# 12302, 1:1000), p-S62 c-Myc (Rabbit, Abcam, Cat# ab51156, 1:1000), p-T58 c-Myc (Rabbit, Abcam, Cat# ab185655, 1:1000), Myc-tag (Mouse, Cell Signaling, Cat# 92013, 1:1000), DYKDDDDK-Tag (Mouse, Thermo Fisher, Cat# MA1-91878, 1:1000), Phosphor-Ser/Thr (Rabbit, Abcam; Cat#ab117253, 1:1000), p-S1525 TOP2A (Cedarlanelabs; Cat#E-AB-21933, 1:1000), TOP2A (Proteintech; Cat#20233-1-AP, 1:1000), Chk1 (Rabbit, Proteintech; Cat#25887-1-AP, 1:1000), Cdc2 (Rabbit, Cell Signaling; Cat#9116T, 1:1000), p-T14 Cdc2 (Rabbit, Cell Signaling; Cat#2543S, 1:1000), SRPK1 (Rabbit, BD biosciences; Cat#611072, 1:1000).

For immunofluorescence experiments, we used antibodies to Nestin (mouse, Abcam, ab22035, 1:500), Ki67 (Rabbit, Thermo Sci, clone SP6, 1:500), BrdU (Mouse, BD Bioscience 347580; Abcam, ab6326, 1:500), Cleaved Caspase 3 (Rabbit, Cell Signaling, 9661, 1:500), c-Myc (Mouse, Cell Signaling; Cat#5605S, 1:500), γH2A.X (Rabbit, Cell Signaling, Cat# 9718S, 1:500), p53 (Mouse, Cell Signaling, Cat# 2524S, 1:500), HA-Tag (Mouse, Cell Signaling; Cat#2367, 1:500), GFAP (Goat, Santa Cruz, sc-6170, 1:500).

Secondary antibodies (donkey anti-rabbit IgG Alexa Fluor 488, Jackson ImmunoResearch, Cat#711-545-152, 1:500; donkey anti-mouse IgG Alexa Fluor 488, Jackson ImmunoResearch, Cat#711-545-150, 1:500; donkey anti-rabbit IgG Alexa Fluor 594, Jackson ImmunoResearch, Cat#711-585-152, 1:500; donkey anti-mouse IgG Alexa Fluor 494, Jackson ImmunoResearch, Cat#711-585-150, 1:500), Biotinylated goat anti-mouse IgG antibody (Vector Laboratories, BA-9200, 1:500).

Validation

All primary antibodies for the the species were validated according to the manufacturer's website with citations of previous publications. Validation data/citations can be found on the manufacture website by searching the antibody catalog number provided in materials and methods section of our manuscript. The primary antibodies were also validated by immunostaining or western blot in the present study.

Eukaryotic cell lines

Policy information about cell lines and Sex and Gender in Research

Cell line source(s)

We obtained the human medulloblastoma cell lines D425, D458, DAOY, D283, HeLa, U2OS, and HEK293T cells lines from ATCC. MB-004 cells were provided by Dr. Martine Roussel. Cerebellar progenitor cells were isolated as described by Nakamura, T. et al. 2016 and confirmed with genotyping.

Authentication

Cell lines were authenticated by short tandem repeat (STR) profiling

Mycoplasma contamination

All cell lines were tested negative for mycoplasma contamination.

Commonly misidentified lines
(See ICLAC register)

No commonly misidentified cell lines were used.

Animals and other research organisms

Policy information about studies involving animals; ARRIVE guidelines recommended for reporting animal research, and Sex and Gender in Research

Laboratory animals

As reported in Methods section of “Animals” for the information of animal species and strains. The genetic mouse strains used in this study were generated and maintained on a mixed C57Bl/6;CD-1 background. Both male and female mice were used for the present

	study. Ages of animals and animal-derived materials used in the study were reported in corresponding legends. Immunodeficient NOD scid gamma (NSG) mice were provided by Cincinnati Children's Hospital Medical Center (CCHMC) animal core. The NSG mice (8-14 week old) of either sex were used and fed (4 or less mice per cage) in a vivarium. Mice were housed at room temperature (20-23 C) with a 12-h light-dark cycle set with lights on from 06:00 to 18:00 and with humidity between 30-80%.
Wild animals	The study did not involve wild animals.
Reporting on sex	Both male and female animals were used in the study
Field-collected samples	The study did not involve samples collected from the field.
Ethics oversight	The animal studies were approved by the IACUC (Institutional Animal Care and Use Committees) of the Cincinnati Children's Hospital Medical Center, USA.

Note that full information on the approval of the study protocol must also be provided in the manuscript.

Flow Cytometry

Plots

Confirm that:

☒ The axis labels state the marker and fluorochrome used (e.g. CD4-FITC).

☒ The axis scales are clearly visible. Include numbers along axes only for bottom left plot of group (a 'group' is an analysis of identical markers).

☒ All plots are contour plots with outliers or pseudocolor plots.

☒ A numerical value for number of cells or percentage (with statistics) is provided.

Methodology

Sample preparation	For cell cycle analysis, the CycleTEST™ PLUS DNA Reagent Kit (BD 340242) used to stain cell nuclei according to the manufacturer's instructions.
Instrument	BD FACSCanto Flow Cytometer
Software	FlowJo V10
Cell population abundance	The histogram of cell cycle distribution was generated from at least 10,000 events per sample.
Gating strategy	No specific gating strategy was used. DAPI and PI staining was used to identify viable cells. DNA contents of the stained cells were analyzed by the flow cytometer using FlowJo v10 software (BD Biosciences).

☐ Tick this box to confirm that a figure exemplifying the gating strategy is provided in the Supplementary Information.
